# Main chemical constituents and mechanism of anti‐tumor action of *Solanum nigrum* L

**DOI:** 10.1002/cam4.7314

**Published:** 2024-08-19

**Authors:** Zhen‐duo Zhao, Cheng Hu, Ling Li, Jia‐qi Zhang, Li‐chao Zhang

**Affiliations:** ^1^ Department of Pharmacy, Shanghai Municipal Hospital of Traditional Chinese Medicine Shanghai University of Traditional Chinese Medicine Shanghai China; ^2^ Experiment Center for Science and Technology Shanghai University of Traditional Chinese Medicine Shanghai China; ^3^ Institute of Vascular Anomalies, Shanghai TCM‐Integrated Hospital, Shanghai University of Traditional Chinese Medicine Shanghai China

**Keywords:** active substance, anti‐cancer, plant extracts, *Solanum nigrum* L.

## Abstract

**Objective:**

*Solanum nigrum L.* (SNL) is a natural drugwith diverse bioactive components and multi‐targeted anti‐tumor effects, gaining increasing attention in clinical application.

**Method and Results:**

This paper reviews the studies on SNL by searching academic databases (Google Scholar, PubMed, Science Direct,and Web of Science, among others), analyzing its chemical compositions (alkaloids, saponins, polysaccharides, and polyphenols, among others), andbriefly describes the anti‐tumor mechanisms of the main components.

**Discussion:**

This paper discusses the shortcomings of the current research on SNL and proposes corresponding solutions, providing theoretical support for further research on its biological functions and clinical efficacy.

## Graphical abstract

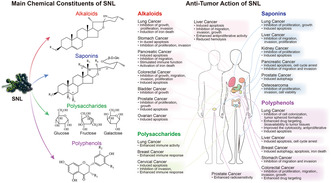

## 1 INTRODUCTION

Cancer is a leading cause of death in humans under 70, with 19 million new incidences and over 9 million cancer‐related deaths in 2020.^1^ Although drug therapy is essential in managing cancer, conventional chemotherapy has significant adverse effects that reduce the quality of life of patients.^2^ Natural medicines are extensively used in clinical applications due to their effectiveness, safety, multi‐component, multi‐targeting, and low toxicity and adverse effects.^3^, ^4^ Therefore, the active ingredients of natural medicines can be extracted and purified to develop anti‐tumor drugs.

*Solanum nigrum* L. (SNL) is a prevalent natural medicine known as Long Kui in China. The extracts of SNL possess tumor‐inhibitory effects; Li et al.^5^ revealed the inhibitory activity of crude SNL extract against C6 high‐grade gliomas in vivo and in vitro. Uen et al.^6^ found that SNL aqueous extract could inhibit glucose uptake of human oral cavity cancer cells (SCC‐4 cells), consequently, their proliferation by inducing mitochondrial apoptosis pathways. Shokrzadeh et al.^7^ found that SNL aqueous extract showed significant cytotoxicity against hepatocellular carcinoma cell lines (HepG2 cells). Lin et al.^8^ found that SNL aqueous inhibits HepG2 cell growth by up‐regulating autophagy‐associated proteins LC3‐I/II, activating the caspase pathway‐induced apoptosis. Moreover, SNL extract exhibited significant cytotoxicity against human breast cancer cells (MCF‐7 cells)^9^ by inhibiting their cell viability through mitochondrial function‐mediated EMT pathway inhibition.^10^ Various active components isolated from SNL could inhibit cancer development by inducing cellular autophagy and inhibiting cell invasion.^11^, ^12^ Therefore, their potential mechanisms of action and related signaling pathways have been extensively studied. Additionally, researchers have focused on combining SNL with other anti‐tumor drugs, preparing novel drug delivery formulations. This paper lists the main SNL chemical components and reviews their anti‐tumor mechanisms to provide a basis for SNL as a potential anti‐tumor drug.

## 2 MAIN CHEMICAL CONSTITUENTS OF SNL

**FIGURE 1:**
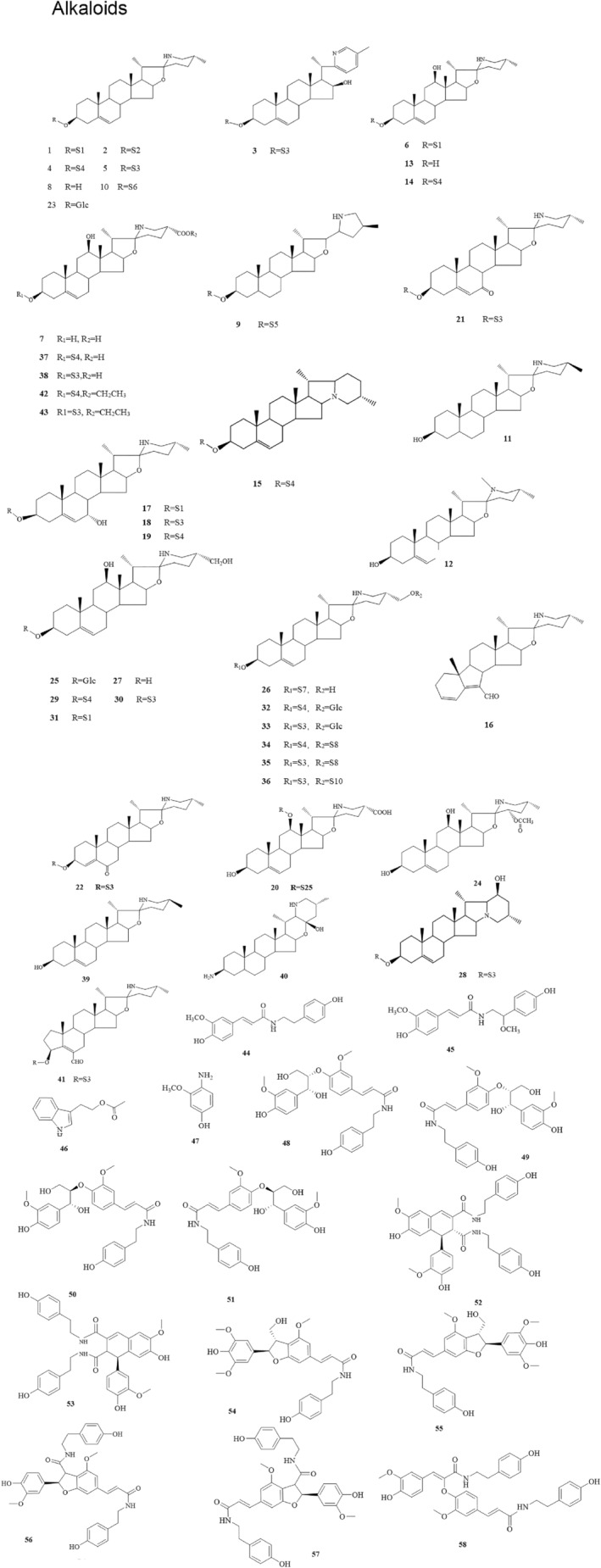
Structural formula for alkaloids.

**FIGURE 2:**
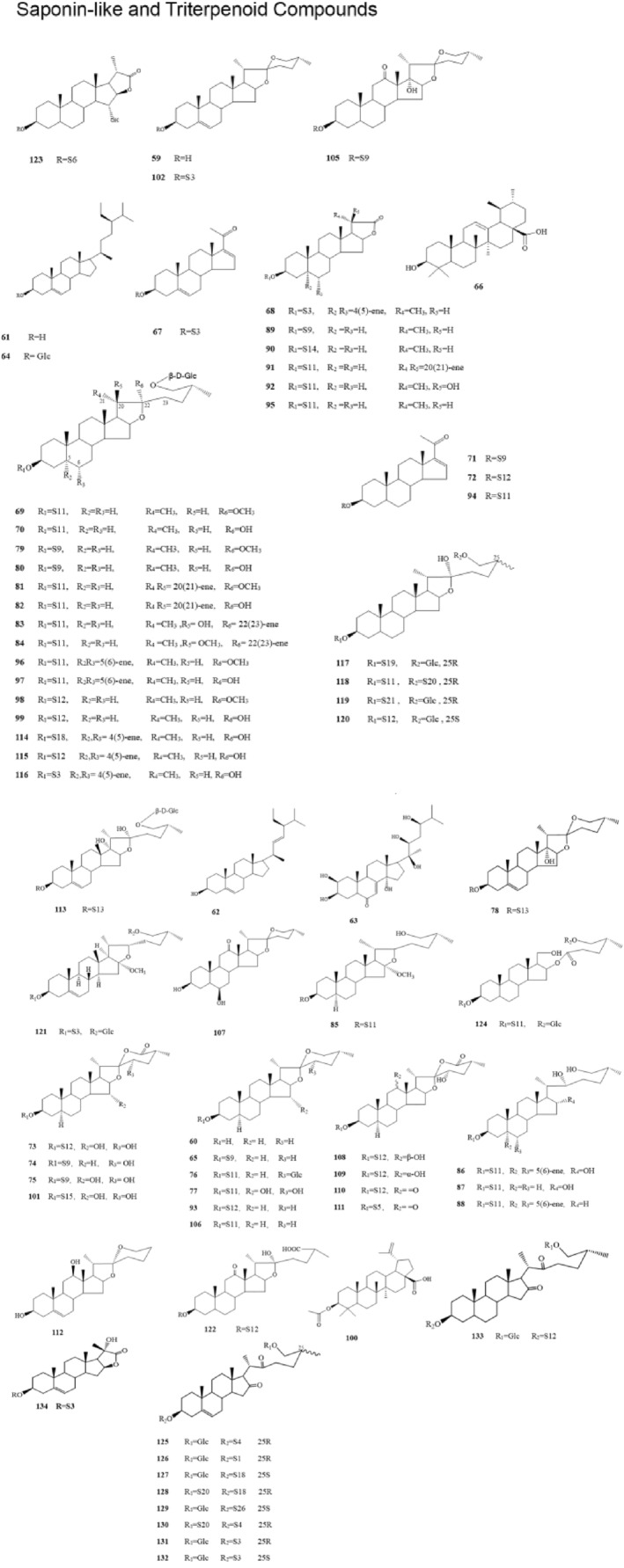
Structural formula for saponin‐like and triterpenoid compounds.

The pharmacodynamic substances that exert anti‐tumor activity in SNL have gained an increasing interest in basic research. The main active components of SNL are steroidal compounds, including steroidal saponins and alkaloids.^13^ In early 1971, Aslanor S.M. found that the main active components in SNL are solasonine (SN), solamargine (SM), and solasodine (SD). In 1982, Japanese scholars^14^ isolated SN and SM from SNL, and later, Gu et al. isolated four new alkaloids (solanine A, 7α‐OH khasianine, 7α‐OH solamargine, and 7α‐OH solasonine). Among them, solanine A showed significant cytotoxicity against the gastric cancer cell line (MGC803 cells), HepG2 cells, and colon cancer cell line (SW480 cells). In 2006, Zhou et al.^15^ isolated 45 compounds from SNL, which included eight alkaloidal steroidal compounds (Table 1; Figure 1A,B, compounds:1‐7, 10, 24, 39‐40) and 38 non‐alkaloidal type steroidal saponins (Table 2; Figure 2A, compounds:65‐99, 104‐106). In 2022, Yang et al.^16^ isolated solalyraine A from SNL with D151 macroporous weakly acidic acrylic cation exchange resin, octadecylsilyl column chromatography, and high‐performance liquid chromatography (HPLC). Other scholars have isolated multiple chemical components from SNL.^17^, ^18^, ^19^, ^20^, ^21^, ^22^, ^23^, ^24^

**TABLE 1:** Alkaloid compounds.

No.	Compound	Molecular formula	Molecular mass	Bibliography
1	β2‐solamargine/khasianine	C_39_H_63_NO_11_	721.9	^15^
2	β2‐solasonine	C_39_H_63_NO_12_	737.9	^15^
3	Solanigroside Q	C_45_H_69_NO_15_	863.5	^15^
4	Solasonine	C_45_H_73_NO_16_	884.1	15, 32
5	Solamargine	C_45_H_73_NO_15_	868.1	^15^
6	Solanigroside P	C_39_H_63_NO_12_	737.9	15, 17
7	(3β,12β,22α,25R)‐3,12‐dihydroxy‐spirosol‐5‐en‐27‐oic acid	C_27_H_41_NO_5_	459.6	^15^
8	Solasodine	C_27_H_43_NO_2_	413.6	^16^
9	Solalyraine A	C_45_H_75_NO_17_	902.1	^16^
10	β1‐solasonine	C_39_H_63_NO_11_	721.9	15, 17
11	Tomatidine	C_27_H_45_NO_2_	415.6	^33^
12	N‐methylsolasodine	C_28_H_45_NO_2_	427.7	15, 34
13	Solanaviol	C_27_H_43_NO_3_	429.7	19, 34
14	12β‐hydroxysolasodine β‐solatrioside	C_45_H_73_NO_17_	900.1	^15^
15	α‐solanine	C_45_ H_73_NO_15_	868.1	^33^
16	Solanine A	C_26_H_38_NO C_27_H_39_NO_2_	409.6	^21^
17	7ɑ‐OH khasianine	C_39_H_63_NO_12_	737.9	^21^
18	7ɑ‐OH solamargine	C_45_H_73_NO_16_	884.1	^21^
19	7ɑ‐OH solasonine	C_45_H_73_NO_17_	900.0	^21^
20	Solaoiacid	C_45_H_71_NO_19_	929.5	^35^
21	(25R)‐22α‐N‐spirosol‐5(6)‐en‐3β‐ol‐7‐oxo‐3‐O‐α‐L‐rhamnopyranosyl‐(1→2)‐[α‐L‐rhamnopyranosyl‐(1→4)]‐β‐D‐glucopyranoside	C_45_H_71_NO_16_	882. 05	^36^
22	(25R)‐22α‐N‐spirosol‐4(5)‐en‐3β‐ol‐6‐oxo‐3‐O‐α‐L‐rhamnopyranosyl‐(1→2)‐[α‐L‐rhamnopyranosyl‐(1→4)]‐β‐D‐glucopyranoside	C_45_H_71_NO_16_	882.05	^36^
23	γ‐solamargine	C_33_H_53_NO_7_	575.79	^17^
24	23‐O‐acetyl‐12β‐hydroxysolasodine	C_29_H_45_NO_5_	487.3	^15^
25	Solanigrine C/12β,27‐dihydroxy solasodine‐3‐O‐β‐D‐glucopyranoside	C_33_H_53_NO_9_	607.8	^37^
26	27‐hydroxysolasodine‐3‐O‐β‐D‐glucopyranosyl‐(1→4)‐α‐L‐rhamnopyranosyl‐(1→2)‐[α‐L‐rhamnopyranosyl‐(1→4)]‐β‐D‐glucopyranoside	C_51_H_83_NO_21_	1045.2	^37^
27	12β,27‐dihydroxy solasodine	C_27_H_43_NO_4_	445.6	^37^
28	Leptinine I	C_45_H_73_NO_15_	867.5	^33^
29	12β,27‐dihydroxysolasodine 3‐O‐α‐L‐rhamnopyranosyl‐(1→2)‐[O‐β‐D‐glucopyranosyl‐(1→3)]‐O‐β‐D‐galactopyranoside	C_45_H_73_NO_18_	916.1	16, 38
30	12β,27‐dihydroxy solasodine β‐chacotrioside	C_45_H_73_NO_17_	900.1	^19^
31	Solanigrine D	C_39_H_63_NO_13_	753.9	^16^
32	Solanigrine G	C_51_H_83_NO_22_	1061.5	^16^
33	Solanigrine H	C_51_H_83_NO_21_	1045.5	^16^
34	Solanigrine E	C_57_H_93_NO_27_	1223.6	^16^
35	Solanigrine F	C_57_H_93_NO_26_	1207.6	^16^
36	Solanigrine I	C_63_H_103_NO_21_	1369	^16^
37	Solanigrine A	C_45_H_71_NO_19_	929.5	^16^
38	Solanigrine B	C_45_H_71_NO_18_	913.5	^16^
39	Tomatidenol	C_27_H_43_NO_2_	413.6	^15^
40	Solanocapsine	C_27_H_46_N_2_O_2_	430.4	^15^
41	(25R)‐22α‐N‐4‐nor‐spirosol‐5(6)‐en‐3β‐ol‐6‐al‐3‐O‐α‐L‐rhamnopyranosyl‐(1 → 2)‐[α‐L‐rhamnopyranosyl‐(1 → 4)]‐β‐D‐glucopyranoside	C_45_H_71_NO_16_	882.1	^36^
42	Solanigrine J	C_47_H_75_NO_19_	957.5	^16^
43	Solanigrine K	C_47_H_75_NO_18_	941.5	^16^
44	N‐*trans*‐feruloyl‐tyramine	C_18_H_19_NO_4_	313.3	^39^
45	(R)‐3‐(4‐hydroxy‐3‐methoxyphenyl)‐N‐[2‐(4‐hydroxyphenyl)‐2‐methoxyethyl]acrylamide	C_19_H_21_NO_5_	343.1	^39^
46	Tryptophol acetate	C_12_H_13_NO_2_	203.1	^39^
47	4‐amino‐3‐methoxyphenol	C_7_H_9_NO_2_	139.2	^39^
48	(7R, 8S)‐1‐(4‐hydroxy‐3‐methoxyphenyl)‐2‐{4‐{2‐[N‐2‐(4‐hydroxyphenyl)ethyl]carbamoylehenyl‐2‐methoxyphenoxyl}}‐1,3‐propanodiolnamed	C_28_H_31_NO_8_	509.2	^40^
49	(7R, 8R)‐1‐(4‐hydroxy‐3‐methoxyphenyl)‐2‐{4‐{2‐[N‐2‐(4‐hydroxyphenyl)ethyl]carbamoylehenyl‐2‐methoxyphenoxyl}}‐1,3‐propanodiolnamed	C_28_H_31_NO_8_	509.2	^40^
50	(7R, 8R)‐1‐(4‐hydroxy‐3‐methoxyphenyl)‐2‐{4‐{2‐[N‐2‐(4‐hydroxyphenyl)ethyl]carbamoylehenyl‐2‐methoxyphenoxyl}}‐1, 3‐propanodiolnamed	C_28_H_31_NO_8_	509.2	^40^
51	(7S,8S)‐1‐(4‐hydroxy‐3‐methoxyphenyl)‐2‐{4‐{2‐[N‐2‐(4‐hydroxyphenyl)ethyl]carbamoylehenyl‐2‐methoxyphenoxyl}}‐1,3‐propanodiolnamed	C_28_H_31_NO_8_	509.2	^40^
52	7′ S,8′R‐7‐hydroxy‐1‐(4‐hydroxy‐3‐methoxyphenyl)‐N^2^, N^3^‐bis(4‐hydroxyphenethyl)‐6‐methoxy‐1,2‐dihydronaphthalene‐2, 3‐dicarboxamide	C_36_H_36_N_2_O_8_	624.7	^40^
53	7′R,8′ S‐7‐hydroxy‐1‐(4‐hydroxy‐3‐methoxyphenyl)‐N^2^, N^3^‐bis(4‐hydroxyphenethyl)‐6‐methoxy‐1,2‐dihydronaphthalene‐2, 3‐dicarboxamide	C_36_H_36_N_2_O_8_	624.7	^40^
54	7′R, 8′ S‐7‐(4‐hydroxy‐3, 5‐dimethoxyphenyl)‐3′‐hydroxymethyl‐1′‐[N‐7″‐(4″‐hydrxyphenyl)ethyl] carbamoylethenyl‐3′‐methoxybenzodihydrofuran	C_29_H_31_NO_8_	521.6	^40^
55	7′ S, 8′R‐7‐(4‐hydroxy‐3, 5‐dimethoxyphenyl)‐3′‐hydroxymethyl‐1′‐[N‐7″‐(4″‐hydrxyphenyl)ethyl] carbamoylethenyl‐3′‐methoxybenzodihydrofuran	C_29_H_31_NO_8_	521.6	^40^
56	(7′R, 8′R)‐2‐(4‐Hydroxy‐3‐methoxyphenyl)‐3‐[N‐2‐(4‐hydroxyphenyl)ethyl]carbamoyl‐5‐[N‐2‐(4‐hydroxyphenyl)ethyl]carbamoylethenyl‐7‐methoxybenzodihydrofurn	C_36_H_36_N_2_O_8_	624.7	^40^
57	(7′ S, 8′ S)‐2‐(4‐Hydroxy‐3‐methoxyphenyl)‐3‐[N‐2‐(4‐hydroxyphenyl)ethyl]carbamoyl‐5‐[N‐2‐(4‐hydroxyphenyl)ethyl]carbamoylethenyl‐7‐methoxybenzodihydrofurn	C_36_H_36_N_2_O_8_	624.7	^40^
58	Cannabisin F	C_36_H_36_N_2_O_8_	624.7	^40^

**TABLE 2:** Saponin‐like and triterpenoid compounds.

No.	Compound	Molecular formula	Molecular mass	Bibliography
59	Diosgenin	C_27_H_42_O_3_	414.3	13, 38
60	Tigogenin	C_27_H_44_O_3_	416.3	13, 39
61	β‐Sitosterol	C_29_H_50_O	414.4	13, 39, 41
62	Stigmasterol	C_29_H_48_O	412.4	13, 39
63	Pterosterone	C_27_H_44_O_7_	480.3	13, 39
64	Daucosterol	C_35_H_60_O_6_	576.4	13, 39
65	NigrumninI	C_55_H_90_O_25_	1150.6	15, 42
66	Ursolic acid	C_30_H_48_O_3_	456.7	^39^
67	Hypoglaucin H	C_39_H_60_O_15_	768.4	^15^
68	Dumoside	C_40_H_62_O_16_	798.4	^15^
69	Uttroside A	C_57_H_96_O_28_	1228.6	^15^
70	Uttroside B	C_56_H_94_O_28_	1214.6	^15^
71	Solanigroside A	C_49_H_78_O_24_	1050.5	^15^
72	Solanigroside B	C_45_H_72_O_22_	964.5	^15^
73	Solanigroside C	C_51_H_82_O_23_	1110.5	^15^
74	Solanigroside D	C_55_H_88_O_27_	1180.6	^15^
75	Solanigroside E	C_55_H_88_O_28_	1196.6	^15^
76	Solanigroside F	C_56_H_92_O_28_	1212.6	^15^
77	Solanigroside G	C_50_H_82_O_23_	1050.5	^15^
78	Solanigroside H	C_51_H_82_O_22_	1046.5	^15^
79	Solanigroside I	C_62_H_104_O_31_	1344.7	^15^
80	Solanigroside J	C_61_H_102_O_31_	1330.6	^15^
81	Solanigroside K	C_57_H_94_O_28_	1226.6	^15^
82	Solanigroside L	C_56_H_92_O_28_	1212.6	^15^
83	Solanigroside M	C_56_H_92_O_28_	1212.6	^15^
84	Solanigroside N	C_57_H_94_O_28_	1226.6	^15^
85	Solanigroside O	C_51_H_86_O_23_	1066.6	^15^
86	Solanigroside R	C_50_H_84_O_23_	1052.5	^15^
87	Solanigroside S	C_50_H_86_O_23_	1054.6	^15^
88	Solanigroside T	C_50_H_84_O_22_	1036.6	^15^
89	Solanigroside U	C_50_H_80_O_25_	1080.5	^15^
90	Solanigroside V	C_50_H_80_O_25_	1080.5	^15^
91	Solanigroside W	C_45_H_70_O_22_	962.4	^15^
92	Solanigroside X	C_45_H_72_O_23_	980.4	^15^
93	Tigogenin‐3‐O‐β‐D‐glucopyranosyl‐(1→2)‐O‐[β‐D‐glucopyransyl‐(1→3)]‐O‐β‐D‐glucopyranosyl‐(1→4)‐O‐β‐D‐galactopyranoside	C_51_H_84_O_23_	1064.5	^15^
94	5α‐pregn‐16‐en‐3β‐ol‐20‐one‐lycotetraoside	C_44_H_70_O_21_	934.4	^15^
95	(5α,20S)‐3β,16β‐dihydroxy pregn‐22‐carboxylic acid(22,16)‐lactone‐3‐[O‐β‐D‐glucopyranosyl‐(1→2)]‐O‐[β‐D‐xylopyranosyl‐(1→3)]‐O‐β‐D‐glucopyranosyl‐(1→4)‐O‐β‐D‐galactopyranoside	C_45_H_72_O_22_	964.5	^15^
96	(22α,25R)‐26‐O‐(β‐D‐glucopyranosyl)‐22‐methoxy‐furost‐Δ5‐3β,26‐diol‐3‐O‐β‐D‐glucopyranosyl‐(1→2)‐O‐[β‐D‐xylopyranosyl‐(1→3)]‐O‐β‐D‐glucopyranosyl‐(1→4)‐O‐β‐D‐galactopyranoside	C_56_H_94_O_28_	1226.6	^15^
97	(22α,25R)‐26‐O‐(β‐D‐glucopyranosyl)‐22‐hydroxy‐furost‐Δ5‐3β,26‐diol‐3‐O‐β‐D‐glucopyranosyl‐(1→2)‐O‐[β‐D‐xylopyranosyl‐(1→3)]‐O‐β‐D‐glucopyranosyl‐(1→4)‐O‐β‐D‐galactopyranoside	C_56_H_92_0_28_	1212.6	^15^
98	(5α,22α,25R)‐26‐O‐(β‐D‐glucopyranosyl)‐22‐methoxy‐furostan‐3β,26‐diol‐3‐O‐β‐D‐glucopyranosyl‐(1→2)‐O‐[β‐D‐glucopyranosyl‐(1→3)]‐O‐β‐D‐glucopyranosyl‐(1→4)‐O‐β‐D‐galactopyranoside	C_58_H_98_O_29_	1258.6	15, 41
99	(5α,22α,25R)‐26‐O‐(β‐D‐glucopyranosyl)‐22‐hydroxy‐furost‐3β,26‐diol‐3‐O‐β‐D‐glucopyranosyl‐(1→2)‐O‐[β‐D‐glucopyranosy‐(1→3)]‐O‐β‐D‐glucopyranosyl‐(1→4)‐O‐β‐D‐galactopyranoside	C_57_H_96_O_29_	1244.6	15, 41
100	3‐o‐acetylbetulinic acid	C_32_H_50_O_4_	498.4	
101	Soladulcoside a	C_39_H_62_O_15_	770.4	^23^
102	Dioscin	C_45_H_72_O_16_	868.5	^23^
103	Asperin	C_51_H_82_O_20_	1014.5	^23^
104	Uttronin b	C_39_H_62_O_12_	722.1	15, 41, 43
105	Nigrumnin II	C_55_H_88_O_27_	1180.5	15, 42
106	Uttronin A/Degalactotigonin	C_50_H_82_O_22_	1034.5	15, 23, 43
107	12‐keto‐porrigenin	C_27_H_42_O_5_	446.3	^39^
108	Solanigroside Y1	C_51_H_82_O_26_	1110.5	^41^
109	Solanigroside Y2	C_51_H_82_O_26_	1110.5	^41^
110	Solanigroside Y3	C_51_H_80_O_26_	1108.5	^41^
111	Solanigroside Y4	C_45_H_70_O_21_	946.4	^41^
112	Spirost‐5‐ene‐3β,12β‐diol	C_26_H_40_O_4_	416.3	^17^
113	Solanigroside Y5	C_57_H_94_O_28_	1226.6	^41^
114	Solanigroside Y6	C_57_H_94_O_27_	1210.6	^41^
115	(25R)‐26‐O‐β‐D‐glucopyranosylfurost‐5(6)‐ene‐3β,22α,26‐triol‐3‐O‐β‐D‐glucopyranosyl‐(1→2)‐[β‐D‐glucopyranosyl‐(1→3)]‐β‐D‐glucopyranosyl‐(1→4)‐β‐D‐galactopyranoside	C_57_H_94_O_29_	1242.6	^41^
116	(25R)‐26‐O‐β‐D‐glucopyranosylfurost‐5(6)‐ene‐3β,22α,26‐triol‐3‐O‐α‐L‐rhamnopyranosyl‐(1→2)‐[α‐L‐rhamnopyranosyl‐(1→4)]‐β‐D‐glucopyranoside	C_51_H_84_O_22_	1048.6	^41^
117	Solanigroside Y7	C_63_H_106_O_34_	1406.7	^44^
118	Solanigroside Y8	C_62_H_104_O_33_	1376.7	^44^
119	Solanigroside Y9	C_62_H_104_O_33_	1376.7	^44^
120	(25S)‐26‐O‐β‐D‐glucopyranosyl‐5α‐furost‐3β,22α,26‐triol‐3‐O‐β‐D‐glucopyranosyl‐(1→2)‐[β‐D‐glucopyranosyl‐(1→3)]‐β‐D‐glucopyranosyl‐(1→4)‐β‐D‐galactopyranoside	C_57_H_93_O_29_	1242.33	^42^
121	(25R)‐26‐O‐β‐D‐glucopyranosylfurost‐5(6)‐ene‐16α‐methoxy‐3β,26‐diol‐3‐O‐α‐L‐rhamnopyranosyl‐(1→2)‐[α‐L‐rhamnopyranosyl‐(1→4)]‐β‐D‐glucopyranoside	C_52_H_86_O_22_	1062.6	^44^
122	3β,15α,16β‐trihydroxypregnane‐20‐carboxylic	C_51_H_82_O_26_	1110.5	^40^
123	Acid‐16,22‐lactone 3‐O‐α‐L‐rhamnopyranosyl‐(1→2)‐O‐β‐D‐galactopyranoside	C_34_H_54_O_13_	670.8	^36^
124	Nigroside A	C_56_H_94_O_29_	1230.6	44, 45
125	(25R)‐26‐O‐β‐D‐glucopyranosyl‐cholest‐5(6)‐en‐3β,26‐diol‐16,22‐dione‐3‐O‐α‐L‐rhamnopyranosyl‐(1→2)‐[β‐D‐glucopyranosyl‐(1→3)]‐β‐D‐galactopyranoside	C_51_H_82_O_23_	1062.6	^46^
126	(25R)‐26‐O‐β‐D‐glucopyranosyl‐cholest‐5(6)‐en‐3β,26‐diol‐16,22‐dione‐3‐O‐α‐L‐rhamnopyranosyl‐(1→4)‐β‐D‐glucopyranoside	C_45_H_72_O_18_	900.5	^46^
127	(25S)‐26‐O‐β‐D‐glucopyranosyl‐cholest‐5(6)‐en‐3β,26‐diol‐16,22‐dione‐3‐O‐α‐L‐rhamnopyranosyl‐(1→2)‐[α‐L‐rhamnopyranosyl‐(1→4)]‐[β‐D‐glucopyranosyl‐(1→6)]‐β‐D‐glucopyranoside	C_57_H_92_O_27_	1208.6	^46^
128	(25R)‐26‐O‐β‐D‐glucopyranosyl‐(1→2)‐β‐D‐glucopyranosyl‐cholest‐5(6)‐en‐3β,26‐diol‐16,22‐dione‐3‐O‐α‐L‐rhamnopyranosyl‐(1→2)‐[α‐L‐rhamnopyranosyl‐(1→4)]‐[β‐D‐glucopyranosyl‐(1→6)]‐β‐D‐glucopyranoside	C_63_H_102_O_32_	1370.7	^46^
129	(25S)‐26‐O‐β‐D‐glucopyranosyl‐cholest‐5(6)‐en‐3β,26‐diol‐16,22‐dione‐3‐O‐β‐D‐glucopyranosyl‐(1→6)‐β‐D‐glucopyranosyl‐(1→3)‐[α‐L‐rhamnopyranosyl‐(1→2)]‐β‐D‐galactopyranoside	C_57_H_92_O_28_	1224.6	^46^
130	(25R)‐26‐O‐β‐D‐glucopyranosyl‐(1→2)‐β‐D‐glucopyranosyl‐cholest‐5(6)‐en‐3β,26‐diol‐16,22‐dione‐3‐O‐α‐L‐rhamnopyranosyl‐(1→2)‐[β‐D‐glucopyranosyl‐(1→3)]‐β‐D‐galactopyranoside	C_57_H_92_O_28_	1224.6	^46^
131	(25R)‐26‐O‐β‐D‐glucopyranosyl‐cholest‐5(6)‐en‐3β,26‐diol‐16,22‐dione‐3‐O‐α‐L‐rhamnopyranosyl‐(1→2)‐[α‐L‐rhamnopyranosyl‐(1→4)]‐β‐D‐glucopyranoside	C_51_H_82_O_22_	1047.2	^46^
132	(25S)‐26‐O‐β‐D‐glucopyranosyl‐cholest‐5(6)‐en‐3β,26‐diol‐16,22‐dione‐3‐O‐α‐L‐rhamnopyranosyl‐(1→2)‐[α‐L‐rhamnopyranosyl‐(1→4)]‐β‐D‐glucopyranoside	C_51_H_82_O_22_	1047.2	^46^
133	(25R)‐26‐O‐β‐D‐glucopyranosyl‐cholest‐5α‐3β,26‐diol‐16,22‐dione‐3‐O‐β‐D‐glucopyranosyl‐(1→2)‐[β‐D‐glucopyranosyl‐(1→3)]‐β‐D‐glucopyranosyl‐(1→4)‐β‐D‐galactopyranoside	C_57_H_94_O_29_	1242.6	^46^
134	(20S)‐3β,16α,20‐trihydroxy‐pregn‐5‐en‐20‐carboxylic acid (22,16)‐lactone‐3‐O‐α‐L‐rhamnopyranosyl‐(1→2)‐[α‐L‐rhamnopyranosyl‐(1→4)]‐α‐D‐glucopyranoside	C_40_H_62_O_17_	814.4	^46^

**FIGURE 3:**
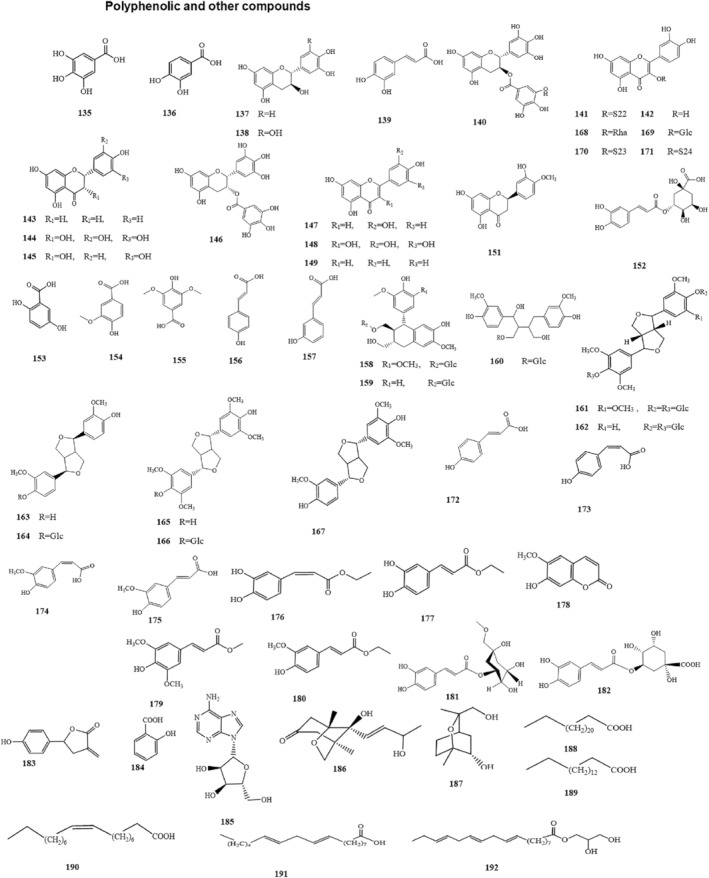
Structural formula for polyphenolic and other compounds.

**FIGURE 4:**
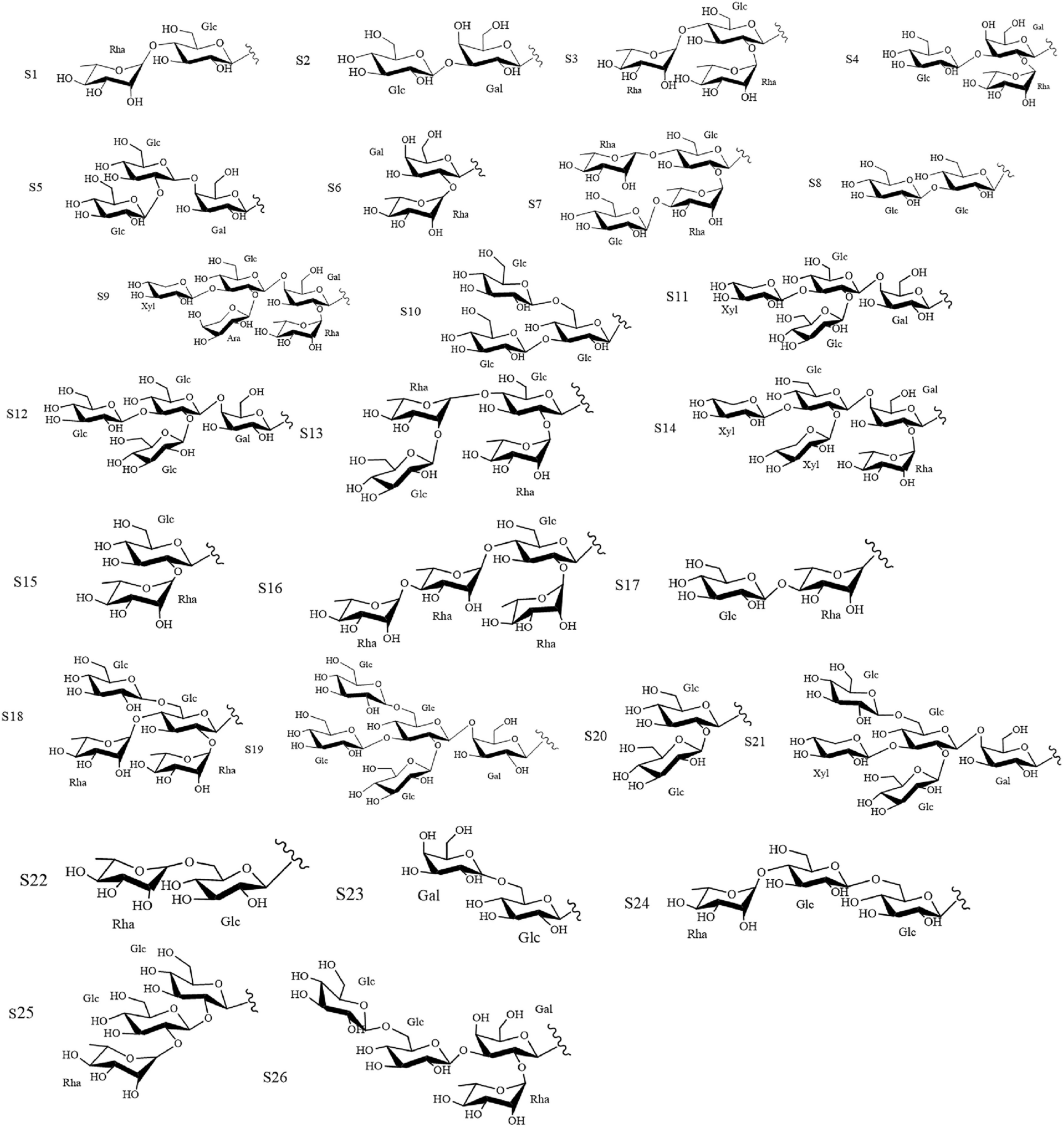
Structural formula for substituent group.

Moreover, SNL contains polysaccharides and polyphenols. Xiao et al.^25^, ^26^, ^27^ isolated the polysaccharide components SNL‐1/2/3/4 from SNL by water extraction (Table 3, compounds: a‐d). Liu et al.^28^ isolated the alkaline‐soluble polysaccharides SNLBP (Table 3, compounds: e) and confirmed it as a single component. Li et al.^29^ isolated SNL‐P from SNL by ethanol subsiding method and used DEAE‐cellulose ion exchange chromatography to isolate SNL‐P1/2/3 (Table 3, compounds: f‐h). SNL‐P1 was further purified to obtain SNL‐P1a/b/c by gel permeation chromatography. Yang et al.^30^ extracted the polyphenolic extracts of SNL (SNPE) from SNL and identified its nine main components as gallic acid (GA), protocatechuic acid, caffeic acid, catechin, gallocatechin, gallocatechin gallate, rutin, quercetin, and naringenin (Table 4; Figures 3 and 4, compounds: 135‐143). Huang et al.^31^ identified various polyphenolic compounds(Table 4; Figures 3 and 4, compounds:135‐157) from SNL by HPLC.

**TABLE 3:** Polysaccharide compounds.

Number	Compound	Ingredients	Bibliography
a	SNL1	L‐Rhamnose, D‐xylose, L‐Arabinose and D‐glucose. The composition molar ratio is 4.9:1:2.4:13	25, 26, 27
b	SNL2	L‐Arabinose and D‐glucose. The composition molar ratio is 1:13.3	25, 26, 27
c	SNL3	The composition percentages are D‐Xylose (75.7%), D‐Mannose (9.2%), D‐glucose (4.9%), D‐Galactose (10.2%)	25, 26, 27
d	SNL4	The composition percentages are D‐Xylose (89.4%), D‐Mannose (1.7%), D‐Galactose (8.9%)	25, 26, 27
e	SNLBP	The composition percentages are D‐Xylose (82.2%), D‐Mannose (7.4%), D‐glucose (3.8%), and D‐Galactose (6.6%)	^28^
f	SNFP‐1‐1	D‐glucose, D‐Galacturonic acid, α‐L‐Rhamnopyranose, L‐Arabinose, D‐Galactose. The molar percentages are 52.01:1.36:13.87:4.32:28.43	^29^
g	SNFP‐2‐1	D‐glucose, L‐Arabinose, D‐Mannose, D‐Galactose. The molar percentages are 27.79:16.72:1.6:53.88	^29^
h	SNFP‐3‐1	D‐glucose, L‐Arabinose, D‐Mannose, D‐Xylose, D‐Galactose. The molar percentages are 67.89:9.64:3.2:1.87:17.39	^29^

**TABLE 4:** Polyphenolic and other compounds.

No.	Compound	Molecular formula	Molecular mass	Bibliography
135	Gallic acid	C_7_H_6_O_5_	170.1	30, 31
136	Protocatechuic acid	C_7_H_6_O_4_	154.1	30, 31
137	Catechin	C_15_H_14_O_6_	290.2	30, 31
138	Gallocatechin	C_15_H_14_O_7_	306.2	30, 31
139	Caffeic acid	C_9_H_8_O_4_	180.1	30, 31
140	Gallocatechin gallate	C_22_H_18_O_11_	458.4	30, 31
141	Rutin	C_27_H_30_O_16_	610.5	30, 31
142	Quercetin	C_15_H_10_O_7_	302.2	30, 31
143	Naringenin	C_15_H_12_O_5_	272.2	30, 31
144	Epigallocatechin	C_15_H_14_O_7_	306.2	^31^
145	Epicatechin	C_15_H_14_O_6_	290.2	^31^
146	Epigallocatechin gallate	C_22_H_18_O_11_	458.4	^31^
147	Luteolin	C_15_H_10_O_6_	286.2	^31^
148	Myricetin	C_15_H_10_O_8_	318.2	^31^
149	Apigenin	C_15_H_10_O_5_	270.2	^31^
150	Kaempferol	C_15_H_10_O_6_	286.2	^31^
151	Hesperetin	C_16_H_14_O_6_	302.2	^31^
152	Chlorogenic acid	C_16_H_18_O_9_	354.3	^31^
153	Gentisic acid	C_7_H_6_O_4_	154.1	^31^
154	Vanillic acid	C_8_H_8_O_4_	168.1	^31^
155	Syringic acid	C_9_H_10_O_5_	198.1	^31^
156	p‐Coumaric acid	C_9_H_8_O_3_	164.1	^31^
157	3‐Hydroxycinnamic acid	C_9_H_8_O_3_	164.1	^31^
158	(−)‐5'‐methoxyisolariciresinol‐3α‐O‐β‐D‐glucopyranoside	C_27_H_36_O_12_	552.2	^36^
159	(+)‐isolariciresinol‐3α‐O‐β‐D‐glucopyranoside	C_26_H_34_O_11_	522.2	^36^
160	Cinnacassoside A	C_26_H_36_O_12_	540.2	^36^
161	Acanthoside D	C_34_H_46_O_18_	742.3	^36^
162	(+)‐medioresonol‐di‐O‐β‐D‐glucopyranoside	C_33_H_44_O_17_	712.3	^36^
163	(+)‐pinoresinol	C_20_H_22_O_6_	358.4	^47^
164	(+)‐pinoresinol‐β‐D‐glucoside	C_26_H_32_O_11_	520.5	^48^
165	(+)‐syringaresinol	C_22_H_26_O_8_	418.4	^47^
166	Syringaresinol‐4‐O‐β‐D‐glucopyranoside	C_28_H_36_O_13_	580.6	^48^
167	(+)‐medioresinol	C_21_H_24_O_7_	388.4	^47^
168	Quercitrin	C_21_H_20_O_11_	448.4	^48^
169	Isoquercitrin	C_21_H_20_O_12_	464.4	^48^
170	Quercetin‐3‐O‐β‐D‐Galactopyranosyl‐(1→6)‐β‐D‐glucopyranoside	C_27_H_30_O_17_	626.5	^36^
171	Quercetin‐3‐O‐α‐L‐Rhamanopyranosyl‐(1→4)‐O‐β‐D‐glucopyranosyl‐(1→6)‐O‐β‐D‐glucopyranoside	C_33_H_40_O_21_	772.7	^49^
172	Crans‐p‐Hydroxycinnamic acid	C_9_H_8_O_3_	164.2	^51^
173	Cis‐4‐Hydroxycinnamic acid	C_9_H_8_O_3_	164.2	^36^
174	Cis‐Ferulic acid	C_10_H_10_O_4_	194.2	^50^
175	Trans‐Ferulic acid	C_10_H_10_O_4_	194.2	^50^
176	Ethyl caffeate	C_11_H_12_O_4_	208.2	^50^
177	Ethyl 3,4‐dihydroxycinnamate	C_11_H_12_O_4_	208.2	^50^
178	Scopoletin	C_10_H_8_O_4_	192.2	^47^
179	Methyl sinapate	C_12_H_14_O_5_	238.2	^39^
180	Ethyl 4‐hydroxy‐3‐Methoxycinnamate	C_12_H_14_O_4_	222.2	^39^
181	3‐caffeoylquinic acid methyl ester	C_17_H_22_O_8_	354.4	^36^
182	Chlorogenic acid	C_16_H_18_O_9_	354.3	^39^
183	4‐(4‐hydroxyphenyl)‐2‐methylenebutyrolactone	C_11_H_10_O_3_	190.2	^50^
184	Salicylic acid	C_7_H_6_O_3_	138.1	^51^
185	Adenosine	C_10_H_13_N_5_O_4_	267.2	^48^
186	Drummondol	C_13_H_20_O_4_	240.3	^39^
187	2α,9‐dihydroxy‐1,8‐cineole	C_10_H_18_O_3_	186.25	^39^
188	Tetracosanoic acid	C_24_H_48_O_2_	368.6	^47^
189	Palmitic acid	C_16_H_32_O_2_	256.42	^51^
190	Oleic acid	C_18_H_34_O_2_	282.5	^51^
191	Linoleic acid	C_18_H_32_O_2_	280.45	^51^
192	1‐monolinolenin	C_21_H_36_O_4_	352.51	^52^

## 3 ALKALOIDS

Alkaloids, as one of the main components isolated from SNL, have received extensive attention in the study of their anti‐tumor effects. Haroon Khan et al. summarize the mechanism of action by which alkaloids exert their anticancer potential by inducing cell cycle arrest^53^; Shailima Rampogu et al. summarized the role of alkaloids isolated from natural plants in breast cancer treatment^54^; Arijit Mondal et al. provide an overview of alkaloids with anticancer effects and summarize their mechanisms of action^55^; Caiyan Liu et al. outlined the important role played by alkaloids isolated from traditional Chinese medicines in the treatment of hepatocellular carcinoma; Prasanta Dey et al. provide an overview of the anticancer mechanisms of alkaloidal constituents and make recommendations for the future use of alkaloidal constituents in cancer therapy.^56^ This part mainly describes the anticancer mechanism of action of alkaloid constituents fractionated from SNL. In this article, we will discuss the effect of various alkaloids derived from SNL on various cancers (Table 5).

**TABLE 5:** Anti‐tumor mechanism of alkaloid components.

Cancer	Compound	Cell/animal	Mechanisms	Bibliography
Lung cancer	Solamargine	A549 cells, H1299 cells	Inactivation of PI3K/AKT and down‐regulation of SP1 and p65 expression inhibited EP4 expression	^58^
		A549 cells, H1299 cells	Inhibition of EP4 protein expression down‐regulated ERK1/2 phosphorylation, which in turn led to decreased protein expression levels of DNMT1 and c‐Jun	^59^
		A549 cells, PC9 cells	Inhibition of STAT3 phosphorylation, up‐regulation of FOXO3a protein levels, reduction of SP1 protein expression and inhibition of IGFBP1 expression levels	^60^
		SMH1650 cells, A549 cells	Promotes phosphorylation of p38‐MAPK, inhibits phosphorylation and protein expression of Stat3, and upregulates protein expression of Stat3 downstream effector p21	^61^
		A549 cells, PC9 cells	Increased expression of miR‐214‐3p and inhibition of miR‐214‐3p downstream target gene PDPK1 by binding to HOTAIR	^62^
	Solasodine	A549 cells	Inhibition of miR‐21 expression leads to increased RECK expression, which inhibits mRNA expression of MMP‐2, ‐9, and EMMPRIN	^63^
	Solasonine	Calu‐1 cells, A549 cells	Inhibited expression of GPX4, SLC7A11, disrupted cellular redox homeostasis, induced amplification of mitochondrial ROS and hyperpolarization of mitochondrial membrane potential (MMP), induced cellular iron death	^64^
Liver cancer	Tomatine	HepG2 cells, immunodeficient NSG mice	Down‐regulation of Bcl‐2 and Bcl‐XL expression and concomitant up‐regulation of Bax and Bad expression activates the caspase apoptosis signaling pathway	^68^
	Solamargine	HepG2 cells	Down‐regulation of MMP‐2 and MMP‐9 expression and activity	^69^
		HepG2 cells	Blocking the EMT process	^70^
		HepG2 cells, HepRG cells	Inhibition of GSS and GPX4 expression induces disruption of the GSH redox system and activates iron death	^71^
		SMMC‐7721 cells, HepG2 cells	Up‐regulation of caspase‐3 expression	^72^
		HepG2 cells, Huh‐7 cells	Down‐regulation of the lncRNAs HOTTIP and TUG1 up‐regulated miR‐4726‐5p, which in turn inhibited the MUC1 protein	^73^
		HepG2 cells	LIF/miR‐192‐5p/CYR61/AKT axis induces autophagy in HepG2 cells	^74^
	Solasonine	HepG2 cells, QGY‐7703 cells	Inhibition of cell proliferation through miR‐375‐3p, CCAT1, SP1 and IRF5 axis	^75^
Gastric cancer	Solamargine	AGS cells, BGC823 cells, SGC7901 cells, HGC27 cells MGC803 cells	Inhibition of ERK 1/2 and p‐ERK1/2 MAPK expression and increased lncNEAT1‐2 expression	^77^
		MGC‐803 cells	Up‐regulation of caspase‐3 expression; up‐regulation of Bax/Bcl‐2 ratio and down‐regulation of mutant p53 protein expression	^17^
	Solasonine	SNU1 cells, SNU5 cells	Upregulation of miR‐486‐5p expression specifically targets phosphatidylinositol 3‐kinase regulatory subunit 1 (PI3KR1)	^78^
		SGC‐7901 cells	Mitochondrial apoptosis pathway	^79^
	Tomatidine	85As2 cells	Inhibition of ISGs expression	^80^
	α‐Tomatine	MGC803 cells, BGC823 cells, SGC7901 cells, SGC7901/DDP cells	Inhibition of PI3K/AKT an	^81^
Pancreatic cancer	Solasodine	SW1990 cells, PANC1 cells, SW1990 Hormonal Mouse Model	Down‐regulates the expression of COX‐2, decreases the phosphorylation of p‐AKT and p‐GSK3β, enhances the expression of caspase‐3 and 9, increases the expression of Bax protein, and decreases the expression of Bcl‐2 protein.	^83^
	Fe3O4‐SM	BxPC‐3 cells	Inhibited the AKT/mTOR signaling pathway	^84^
	Solasonine	PANC‐1 cells, CFPAC‐1 cells	Binding to TFAP2A inhibited its protein expression and blocking TFAP2A binding to the OTUB1 promoter region enhanced ubiquitinated degradation of SLC1A1 and activated iron death	^85^
Colorectal cancer	Solasodine	HCT 116 cells, HT‐29 cells和SW480 cells, BALB/c/nu/nu nude mice	Modulation of the AKT/GSK‐3b/b‐catenin signaling pathway and improvement of nuclear translocation of β‐catenin to regulate downstream genes, with a decrease in the protein levels of Bcl‐2, Bcl‐xl and Caspase‐9 and an increase in the protein levels of Bax, Cleaved‐Caspase‐8, Cleaved‐Caspase‐3 and Cleaved‐PARP 1 in a dose‐dependent manner	^87^
	α‐Tomatine	CT‐26 cell	Regulation of non‐Caspase‐dependent pathways	^88^
		HT‐29 cells	Inhibition of APC gene expression	^89^
		HCT‐116 cells, LoVo cells, SW480 cells, SW620 cells, SW48 cells	Induction of mitochondrial release of AIF and caspase‐independent apoptosis	^90^
Bladder cancer	Solasonine	T24 cells, 5637 cells	Binds to NRP 1 and induces NRP 1 protein degradation, inhibits NRP1/VEGFA/VEGFR2 and NRP1/EGFR complex formation, and inhibits MAPK and PI3K/AKT signaling pathways	^91^
Prostate cancer	Solamargine	PC3 cells, DU145 cells	Inhibition of PI3K/AKT pathway enhances docetaxel sensitivity in cancer cells	^92^
	α‐Tomatine	PC‐3 cells	Inhibition of TNF‐α‐induced Akt and IKK activation and phosphorylation of IκBα reduced NF‐κB p50 and p65 nuclear translocation and inhibited NF‐κB activity	^93^
		PC‐3 cells	Reduction of NF‐κB activity and inhibition of its downstream gene Bcl‐2 expression in cells	^94^
		PC‐3 cells	Inhibition of the PI3K/Akt signaling pathway upregulates the expression of the pro‐apoptotic protein BAD and downregulates the expression of the anti‐apoptotic proteins Bcl‐2 and Bcl‐xL	^95^
Nasopharyngeal carcinoma	Solamargine	HNE2 cells, C666‐1 cells	Interaction of lncRNA CCAT1 and miR7‐5p and suppression of SP1 gene expression	^100^
Melanoma	Solamargine	B16F10 cell line	Inhibition of mitosis in tumor cells	^101^
		WM239 cells, WM115 cells	Triggered the extrinsic mitochondrial death pathway	^102^
		WM239 cells, WM115 cells	Triggered the extrinsic mitochondrial death pathway, Up‐regulation of TNFR1, cytochrome c, cathepsin B and Cleaved‐Caspase‐3 protein expression	^103^
Osteosarcoma	Solamargine	U2OS cells	Activation of the mitochondria‐mediated apoptosis pathway	^105^
Multiple myeloma	Solamargine	ARP‐1 cells, NCI‐H929 cells	Activation of cellular autophagy	^107^
Hypopharyngeal squamous cell carcinoma	Solamargine	FaDU cells	Inhibition of LncRNA HOXA11‐As expression prevented lncRNA HOXA11‐AS from binding miR‐155, which resulted in up‐regulation of miR‐155 expression level, inhibited the expression of c‐Myc, a target gene downstream of miR‐155, and down‐regulated c‐Myc protein and up‐regulated P53 protein expression level	^109^
Leukemia	α‐Tomatine	HL‐60 cells	Interaction between alpha‐tomatine and cholesterol components of cell membranes	^110^
		HL‐60 cells, K562 cells	Promotes the release of AIF from mitochondria into the nucleus and downregulates survivin expression	^111^
Ovarian cancer	Tomatine	SKOV‐3 cells	Regulation of the PI3K/AKT/mTOR signaling pathway	^112^
Human choriocarcinoma	α‐Solanine	JEG‐3 cells. Female thymus‐less nude mice	Inhibition of MMP‐2/9 mRNA expression in JEG‐3 cells reduced MMP‐2 activity	^113^
Bile duct cancer	Solamargine	Human bile duct carcinoma QBC939 cells	Increased protein expression of Bax, Caspase 3, Cleaved‐Caspase 3, Caspase 7, and Cleaved‐PARP, but decreased protein expression of Bcl‐2, XIAP and PARP	^114^
Cervix	Solamargine	Ect1/E6E7‐CRL‐2614, HeLa(CCL‐2), SiHa(HTB‐35), female specific pathogen‐free BALB/c nude mice	Inhibited protein expression of CXCL3, inhibited p‐ERK1/2 expression and down‐regulated protein levels of MMP‐2 and MMP‐9 in cervical cancer cells	^115^
Breast cancer	Solamargine	ZR‐75‐1	Reduces the number of HER2/neu receptors on the cell membrane and enhances sensitivity to the chemotherapeutic drugs methotrexate (MTX), 5‐florouracil (5‐Fu), and cisplatin (CDDP)	^116^
Colloid tumor	Solasonine	Human U87 MG, U251 and U118 MG cells, female nu/nu mice	Modulation of the MAPK signaling pathway, targeting p‐p38 and p‐JNK, inhibition of the NF‐κB signaling pathway, thereby inhibiting the nuclear translocation of the p50p65 subunit of NF‐κB, and consequently inhibiting p65 phosphorylation	^117^
Neuroblastoma	Tomatine and tomatidine	SH‐SY5Y	Activation of the PERK/eIF2α pathway and inhibition of proteasome 20S activity interferes with tumor cell protein homeostasis	^119^

### 3.1 Lung cancer

Lung cancer is highly lethal, with 1.8 million new incidences and 1.6 million mortality cases yearly.^57^ The SM may exert its anti‐lung cancer activity by dose‐dependently down‐regulates Protein kinase B (AKT/PKB) phosphorylation, SP1, NF‐κB subunit p65, and E‐prostanoid receptor 4 (EP4) in lung cancer cell lines (A549 and H1299 cells).^58^ Chen et al.^59^ found that SM treatment of A549 and H1299 cells down‐regulated EP4, up‐regulated ERK1/2 phosphorylation, and decreased DNA methyltransferase 1 (DNMT1) and c‐Jun protein expressions. Meanwhile, exogenous EP4 overexpression inhibited ERK1/2 phosphorylation, whereas exogenous DNMT1 overexpression antagonized the inhibitory effect of SM on c‐Jun protein expression, and the exogenous c‐Jun overexpression blocked the inhibitory effect of SM on lung cancer cells, thus inhibiting ERK1/2 phosphorylation. This suggests that SM down‐regulates ERK1/2 phosphorylation by inhibiting EP4 protein expression, decreasing DNMT1 and c‐Jun protein expressions to inhibits the growth of lung cancer cells.

The SM up‐regulates Forkhead box O3 (FOXO3a) protein while down‐regulating SP1 protein by decreasing Signal Transducer and Activator of Transcription 3 (STAT3) phosphorylation and SP1 protein expression in A549 cells and PC9. Therefore, FOXO3a and SP1, as an upstream signal of Insulin‐like growth factor binding protein 1 (IGFBP1), interacted and inhibited IGFBP1 expression in A549 and PC3 cells, exerting the growth inhibitory effect of SM on lung cancer cells.^60^ Zhou et al.^61^ found that SM inhibits proliferation and induces apoptosis in A549 and SMH1650 lung cancer cells. This was achieved by up‐regulating p38‐MAPK phosphorylation while down‐regulating STAT3 phosphorylation and protein, eventually up‐regulating the p21 protein, the downstream effector of STAT3. Tang et al.^62^ found that SM treatment of A549 and PC9 cells down‐regulated long non‐coding RNA HOX transcript antisense RNA (lncRNA HOTAIR) expression and promoter activity while significantly overexpressing miR‐214‐3p. Moreover, they have revealed that microRNA (miR)‐214‐3p had a binding site in the 3'‐UTR region of lncRNA HOTAIR. Therefore, miR‐214‐3p binding to HOTAIR suppressed PDPK1, the downstream target gene of miR‐214‐3p. This may be the mechanism of action of SM to inhibit NSCLC cell proliferation.

SD could inhibit A549 cell invasion by regulating the mitochondrial membrane potential (MMP) pathway and exerting an anti‐lung cancer effect.^63^ The mechanism of action is that SD down‐regulates miR‐21 while up‐regulating Reversion Inducing Cysteine Rich Protein With Kazal Motifs (RECK), a negative MMP regulator, thus inhibiting MMP‐2/9 and EMMPRIN mRNA expression.

Zeng et al.^64^ found that SN may exert anti‐tumor effects by inducing cellular iron death by causing redox imbalance and mitochondrial dysfunction. A dose‐dependent increase in Fe^2+^ level was observed after SN treatment of Calu‐1 and A549 cells, implying that SN may have induced iron death. The mechanism of action is achieved through SN inhibiting the antioxidant enzyme glutathione peroxidase 4 (GPX4), expressing SLC7A11, the cystine/glutamate transporter protein, disrupting cellular redox homeostasis, and inducing mitochondrial ROS amplification and MMP hyperpolarization.

### 3.2 Liver cancer

Hepatocellular carcinoma is a common and fatal malignancy worldwide, and using natural compounds represents a viable therapeutic option.^65^ Components including SM, SN, and α‐tomatine are cytotoxic to hepatocellular carcinoma cells.^66^, ^67^ Echeverría et al.^68^ found that tomatine obtained from SNL induces apoptosis in the human HepG2 cells, and intraperitoneal injections of 5 and 20 mg/kg of tomatine significantly reduced the tumor size in xenograft tumor model mice. The potential mechanism is that tomatine induces HepG2 cell apoptosis by down‐regulating Bcl‐2/xl via the p53 pathway while up‐regulating Bax and Bad, activating the caspase apoptosis signaling pathway.

SM could down‐regulate MMP‐2/9 expression, blocking the EMT pathway and inhibiting HepG2 cell migration and invasion.^69^, ^70^ Additionally, SM down‐regulates the mRNA and protein levels of the iron death regulator, GSS, and the antioxidant enzyme, GPX4, to disrupt the GPX4‐induced GSH redox system, promoting the iron death of HepG2/RG cells.^71^ Ding et al.^72^ found that SM could up‐regulate caspase‐3, induce the apoptosis of hepatocellular carcinoma cell lines (SMMC‐7721 and HepG2 cells), and cause cell cycle arrest in the G2/M phase. Moreover, SM can exert anti‐tumor effects by regulating non‐coding RNAs. Tang et al.^73^ found that miR‐4726‐5p has binding sites with lncRNA HOTTIP and TUG1 and can bind to the 3'‐UTR region of the MUC1 protein to regulate its expression. SM treatment in human hepatocellular carcinoma cell lines (HepG2 and Huh‐7 cells) reduced MUC1 protein expression and lncRNA HOTTIP and TUG1 while overexpressing miR‐4726‐5p. This suggests that SM inhibits tumor cell proliferation by regulating the HOTTIP‐TUG1/miR‐4726‐5p/MUC1 signaling pathway. Additionally, SM induces HepG2 cell autophagy and apoptosis via the LIF/miR‐192‐5p/CYR61/AKT axis. Moreover, SM impedes hepatocellular carcinoma invasion by decreasing M2 macrophage polarization and inhibiting the EMT pathway via the LIF/p‐STAT3 pathway.^74^

Liu et al.^75^ found that SN inhibited the growth of hepatocellular carcinoma cells (HepG2 and QGY‐7703 cells) in a time‐ and dose‐dependent manner, with IC50 values of 37.70, 33.88, 35.48 μM and 29.17, 31.83, 35.01 μM at 24, 48, and 72 h, respectively. The SN treatment of HepG2 and QGY‐7703 cells overexpressed miR‐375‐3p. miR‐375‐3p inhibited Colon cancer‐associated transcript‐1 (CCAT1) expression by binding to the 3'‐UTR region of lncRNA CCAT1. Additionally, miR‐375‐3p and CCAT1 acted as upstream factors to inhibit the protein expression of transcription factor SP1, inhibiting IRF5 transcriptional regulation. SN regulated miR‐375‐3p and CCAT1 expression through an IFR5 feedback mechanism. Altogether, SN may exert anti‐tumor effects through miR‐375‐3p/CCAT1/SP1/IRF5 axis.

Nepal et al.^76^ loaded α‐tomatine with mesoporous silica nanoparticles and found that this novel delivery formulation enhanced the antiproliferative activity of α‐tomatine against hepatocellular carcinoma cells and reduced hemolysis.

### 3.3 Stomach cancer

Gastric cancer is among the five most common cancers worldwide, accounting for 7.7% of global cancer deaths and ranking fourth, according to global cancer statistics in 2020.^1^ Fu et al.^77^ found that SM inhibited ERK1/2 phosphorylation and induced lncRNA PINT and NEAT1 expression, inducing apoptosis of gastric cancer cells (SGC7901 and BGC823 cells). Additionally, SM up‐regulates caspase‐3 and Bax/Bcl‐2 ratio while down‐regulating mutant p53 protein in human gastric cancer cells (MGC‐803) in a dose‐dependent manner, inducing apoptosis.^17^

SN inhibits the proliferation of human gastric cancer cell lines (SNU1/5) with IC50 values of 10 and 12.5 μM, respectively. This may be related to the fact that SN upregulates miR‐486‐5p and targets the 3'‐UTR region of PI3KR1.^78^ Li et al.^79^ found that SN dose‐dependently inhibited SGC7901 cell proliferation in vitro with an IC50 of 18 μM by down‐regulating the apoptotic pathway key protein expression, Bcl‐2/xL, inducing apoptosis.

Fujimaki et al.^80^ found that tomatidine inhibits gastric cancer cell proliferation by inhibiting type I interferon‐stimulated genes (ISGs) expression and tumor growth in xenograft tumor model mice. Zhang et al.^81^ found α‐tomatine can inhibit the PI3K/AKT and MAPK signaling pathways inhibit MGC803, BGC823, SGC7901, and SGC7901/DDP cell proliferation, and regulate the transcript levels of key regulators in the EMT pathway to inhibit cell invasion and attenuate the resistance of gastric cancer cells to cisplatin.

### 3.4 Pancreatic cancer

Pancreatic cancer has the lowest 5‐year relative survival rate of 6% and is highly lethal.^82^ Fan et al.^83^ found that the in vivo tumor volume of pancreatic cancer cell (SW1990) hormonal mice was significantly reduced after SD low (10 mg/mL), medium (20 mg/mL), and high (40 mg/mL) dose treatments. Meanwhile, human SW1990 cells showed a dose‐dependent up‐regulation of Bax, cytochrome C, and cleaved‐caspase‐9/3 while down‐regulation of Bcl‐2. COX‐2 protein expression and AKT and GSK3β phosphorylation were significantly down‐regulated, indicating that SD could significantly inhibit the Cox‐2/AKT/GSK3β signaling pathway and induce cell apoptosis. Furthermore, the serum levels of tumor necrosis factor α (TNF‐α), interleukin‐2 (IL‐2), and interferon‐γ (IFN‐γ) were significantly increased in SW1990‐loaded mice, suggesting that SD may exert anti‐tumor activity by stimulating the immune function of the body.

SM inhibits the AKT/mTOR signaling pathway, induces apoptosis, and inhibits cell migration in pancreatic cancer cells (BxPC‐3 cells). Additionally, the inhibitory effect of Fe3O4‐SM was more significant than that of SM treatment alone.^84^ Liang et al.^85^ found that SM inhibited the transcriptional regulation of Transcription factor activating enhancer binding protein 2 alpha (TFAP2A) to OTU domain, ubiquitin aldehyde binding 1 (OTUB1) in human pancreatic cancer cells (PANC‐1 and CFPAC‐1), which in turn inhibited the deubiquitination of SLC7A11 proteins by OTUB1, induced SLC7A11 degradation, activating iron death and inhibiting pancreatic cancer cell progression.

### 3.5 Colorectal cancer

The cancer statistics in China and the United States in 2022 reported more than 590,000 new colorectal cancer cases and 300,000 deaths.^86^ Zhuang et al. found^87^ that SD inhibited the growth of colon cancer cell lines (HCT‐116, HT‐29, and SW‐480 cells) with IC50 values of 39.43, 44.56, and 50.09 μM and dose‐dependently blocked colon cancer cells at G2/M phase. SD treatment significantly down‐regulated p‐PI3K, PI3K, p‐AKT, AKT, mTOR, p‐GSK3β, and β‐catenin while up‐regulating GSK3β and p‐β‐catenin, suggesting that SD may inhibit colon cancer cell growth by regulating AKT/GSK3β/β‐catenin signaling pathway. Additionally, SD treatment increased E‐calmodulin and decreased MMP‐2/9/14 at the transcriptional and translational levels, suggesting that SD could inhibit cell invasion and migration via the MMP and EMT pathways. In vivo experiments showed that the tumor volume and weight of the HCT116 cell transplantation tumor model were significantly reduced, confirming the anti‐colorectal cancer effect of SD.

Kim et al.^88^ found that α‐tomatine promoted apoptosis‐inducing factor (AIF) release from mitochondria into the nucleus and down‐regulated survivin expression, inducing apoptosis by a non‐caspase‐dependent pathway. Ishii et al.^89^ found that tomatine inhibited HT‐29 cell proliferation by up‐regulating the APC gene, the Wnt signaling pathway regulator. Rudolf et al.^90^ found that α‐tomatine was cytotoxic to colorectal cancer cells, which may be related to AIF release from mitochondria and caspase‐independent apoptosis.

### 3.6 Bladder cancer

Bladder cancer is common worldwide, with ~570,000 new cases and 270,000 deaths recorded in 2020.^1^ SN inhibits the growth of human bladder cancer cell lines (T24 and 5637 cells) by binding to the NRP1 protein on the cell membrane. This binding induces extracellular NRP1 protein degradation and impedes NRP1/VEGFA/VEGFR2 and NRP1/EGFR complex formation on the cell membrane surface, inhibiting ERK, P38, and MEK1/2 phosphorylation.^91^

### 3.7 Prostate cancer

Prostate cancer is the leading cause of cancer deaths in men worldwide.^1^ Ge et al.^92^ found that SM significantly inhibited the proliferation of human prostate cancer cells (PC3 and DU145 cells), with IC50 values of 3.25 and 4.52 μM, respectively. The levels of AKT phosphorylation were dose‐dependently reduced in PC3 and DU145 cells after 24 h of SM treatment, consistent with the results of the IHC staining analysis of the xenograft model mice after 8 weeks of administration. This suggests that SM inhibits the PI3K/AKT pathway to inhibit prostate cancer cell proliferation. Moreover, SM combined with docetaxel enhanced the antiproliferative effect on prostate cancer. Lee et al.^93^ identified the potential pathway for α‐tomatine to inhibit prostate cancer to be through inhibiting NF‐κB activity and NF‐κB‐dependent expression of anti‐apoptotic proteins (c‐IAP1, c‐IAP2, Bcl‐2/xL, XIAP, and survivin). The mechanism of action is that α‐tomatine inhibits TNF‐α‐induced activation of AKT and Inhibitor of kappa B Kinase (IKK). Additionally, it hinders inhibitor kappa B alpha (IκBα) phosphorylation, reducing the nuclear translocations of NF‐κB p50 and p65. This inhibition of NF‐κB activity ultimately induces cell apoptosis. Huang et al.^94^ found that the combination of α‐tomatine and curcumin inhibited prostate cancer cell growth, which may be related to the reduction of NF‐κB activity and down‐regulation of its downstream gene, Bcl‐2 in cells and significant down‐regulation of AKT and ERK2/3 phosphorylation. Additionally, α‐tomatine, in combination with curcumin, significantly inhibits tumor growth in vivo in xenograft tumor mice. Lee et al.^95^ found that α‐tomatine inhibits the PI3K/AKT signaling pathway, up‐regulates the pro‐apoptotic protein BAD, and down‐regulates the anti‐apoptotic proteins Bcl‐2/xL, as well as having a synergistic effect with paclitaxel to inhibit prostate cancer.

### 3.8 Nasopharyngeal carcinoma

Nasopharyngeal carcinoma is a malignant epithelial tumor of the apical and lateral walls of the nasopharyngeal cavity with known poor treatment and prognosis.^96^ CCAT1 is a lncRNA highly expressed in various cancers,^97^ and miR7‐5p is a tumor‐suppressor miRNA.^98^, ^99^ Wu et al.^100^ found that SM reduced the lncRNA CCAT1 expression and enhanced miR7‐5p expression in nasopharyngeal carcinoma cell lines (HNE2 and C666‐1 cells). Overexpressed miR7‐5p enhanced the inhibitory effect of SM on lncRNA CCAT1, and overexpressed lncRNA CCAT1 inhibited the up‐regulation of miR7‐5p by SM, suggesting that there is an interaction between the two and that it contributes to the inhibition of nasopharyngeal carcinoma cell growth by SM. Additionally, SM dose‐dependently inhibited mRNA expression of SP1, which was reversed by lncRNA CCAT1overexpressio. Meanwhile, miR7‐5p overexpression enhanced the inhibition of SP1 promoter by SM. overexpression of SP1 did not affect lncRNA CCAT1 expression but reversed the effect of SM on miR7‐5p expression and cell growth inhibition in HNE2 and C666‐1 cells. The interaction of lncRNA CCAT1 and miR7‐5p and SP1 gene expression inhibition may be the mechanism of action of SM in suppressing nasopharyngeal carcinoma.

### 3.9 Melanoma

Melanoma is a fatal skin cancer with increasing incidence worldwide.^1^ SM effectively reduces the tumor tissue size and inhibits mitosis in melanoma model mice.^101^ Al Sinani SS et al.^102^ found that SM activated the extrinsic mitochondrial death pathway, up‐regulated TNFR1, cytochrome c, cathepsin B, and cleaved‐caspase‐3 protein, inhibiting the growth of melanoma cells (WM115/239 cells). Additionally, tomatine inhibits tumor angiogenesis by up‐regulating autophagy markers LC3I/II to induce cellular autophagy in melanoma cell lines (hmel‐1 and M3 cells).^103^

### 3.10 Osteosarcoma

Osteosarcoma is the most common primary solid bone malignancy with a high incidence in children and adolescents.^104^ Treatment of osteosarcoma cells (U2OS1 cells) with SM for 2 h increased mRNA and protein expression levels of p53 and Bax, a key downstream protein for p53 signaling, besides up‐regulating caspase‐3/9 protein and a substantial loss of MMP. These data suggest that SM activates the mitochondria‐mediated apoptotic pathway in U2OS cells.^105^

### 3.11 Multiple myeloma

Multiple myeloma (MM) is a hematological malignancy with more than 32,000 patients newly diagnosed with MM each year.^106^ It was found that SM inhibited the viability of MM cell lines (ARP‐1 and NCI‐H929) in a concentration‐ and time‐dependent manner, with IC50 values of 5.36 and 5.23 μM, respectively, the expression levels of Cleaved‐Caspase‐3 and Bax were up‐regulated and Bcl‐2 was down‐regulated after 24 h of SM (5 μM) treatment, indicating that SM induced apoptosis. Moreover, SM treatment lead to the up‐regulation of proteins related to cell death and autophagy in NCI‐H929 cells, and the inhibitory effect of SM on MM cells and apoptosis were attenuated by co‐treatment with the autophagy inhibitor 3‐methyladenine. It was confirmed that activating cellular autophagy to exert anti‐MM effects might be an effective way for SM to exert anti‐tumor effects.^107^

### 3.12 Hypopharyngeal squamous cell carcinoma

Hypopharyngeal squamous cell carcinoma accounts for 3%–5% of head and neck cancers and is highly aggressive.^108^ Meng et al.^109^ found that SM significantly inhibited FaDu cell viability with an IC50 value of 5.17 μM, and in vitro studies found that xenograft tumor mice sustained a significant decrease in tumor volume and weight after continuous daily intravenous injection of SM (5 mg/kg) for 5 weeks. The possible mechanism of action is that SM inhibited the expression of lncRNA HOXA11‐As, preventing it from binding miR‐155. This prevention up‐regulated miR‐155 expression, down‐regulated the downstream target of miR‐155 and c‐Myc proteins, and up‐regulated the P53 protein.

### 3.13 Leukemia

Leukemia is a common hematological tumor with increasing morbidity and mortality.^1^ α‐tomatine induced apoptosis in human leukemia cancer cell lines (HL‐60 cells), which may be closely related to the interaction between α‐tomatine and cell membrane cholesterol components.^110^ Chao et al.^111^ found that α‐tomatine treatment led to a loss of MMP in the human leukemia cancer cell lines (HL‐60 and K562 cells), releasing the AIF from the mitochondria into the nucleus and down‐regulating survivin to exert significant cytotoxic effects and inhibit HL‐60 xenograft tumor growth.

### 3.14 Ovarian cancer

Ovarian cancer is a highly lethal female cancer. Wu et al.^112^ found that tomatine induced apoptosis in ovarian cancer cell lines (SKOV‐3 cells) in a dose‐ and time‐dependent manner and inhibited cell autophagy by down‐regulating the Beclin‐1 protein. Tomatine treatment down‐regulated the PI3K/AKT/mTOR signaling pathway‐related proteins, suggesting that tomatine may play an anti‐tumor role by inhibiting autophagy and promoting apoptosis.

### 3.15 Others

Xenograft experiments showed that α‐solanine inhibited choriocarcinoma cell growth in nude mice. α‐solanine treatment inhibited MMP‐2/9 mRNA expression in human choriocarcinoma cells (JEG‐3 cells) and reduced MMP‐2 activity, suggesting that α‐solanine inhibits tumor cell metastasis. α‐solanine was not detected as the active form of MMP‐2/9 in the cell culture medium, and its role in activating MMPs needs to be further investigated.^113^ SM up‐regulated Bax, caspase 3, cleaved‐caspase 3, caspase 7, and cleaved‐PARP proteins while down‐regulating Bcl‐2, XIAP, and PARP proteins in human cholangiocarcinoma cells (QBC939 cells). Therefore, SM may inhibit QBC939 cell survival by affecting apoptosis‐related proteins.^114^ Furthermore, SM inhibited the proliferation of cervical cancer cells (HeLa and SiHa cells) by modulating the ERK signaling pathway.^115^ SM significantly reduced the number of HER2/neu receptors on the cell membrane of the breast cancer cell line (ZR‐75‐1 cells) and enhanced the sensitivity of breast cancer cells to chemotherapeutic drugs.^116^ SN regulates NF‐κB and MAPK signaling pathways to inhibit p65 phosphorylation to inhibit glioma cell growth.^117^ Lenka et al.^118^ found that α‐tomatine was cytotoxic to human MCF‐7 cells, with no detectable changes in the apoptosis‐related protein expressions. Meanwhile, the binding of tomatine to cholesterol reduced the cytotoxicity. Silva et al.^119^ found that tomatine and tomatidine treatment activated the PERK/eIF2α pathway and inhibited proteasome 20S activity. Therefore the interference with tumor cell protein homeostasis may be responsible for exerting cytotoxicity against the neuroblastoma cell line (SH‐SY5Y).

In summary, SNL has multi‐component, multi‐pathway and multi‐target anticancer characteristics, of which the main active ingredient, solamargine, has the following anticancer mechanisms: induction of cell autophagy and inhibition of cell migration, regulation of Caspase pathway or lncRNA HOXA11‐As/miR‐155/P53 pathway induces apoptosis, regulation of PI3K/AKT pathway or lncRNA CCAT1/miR7‐5p/SP axis inhibits tumor cell growth. Reduces the number of HER2/neu receptors on the cell membrane and enhances sensitivity to the chemotherapeutic drugs. The main anticancer mechanisms of Solasodine include: modulation of the caspase pathway to induce apoptosis, down‐regulation of miR‐21 and MMPs to inhibit tumor cell migration, modulation of the AKT/GSK‐3b/b‐catenin signaling pathway and improvement of nuclear translocation of β‐catenin to induce apoptosis, modulation of the AKT/GSK‐3b/b‐catenin signaling pathway and improvement. The main anti‐cancer mechanisms of Solasodine include. Induction of cellular iron death by disrupting cellular redox homeostasis by inhibiting the expression of GPX 4 and SLC7A11, Inhibition of cell proliferation through miR‐375‐3p/CCAT1/ SP1/IRF5 axis, induction of apoptosis through mitochondrial apoptosis pathway, enhanced ubiquitinated degradation of SLC1A1 to activated iron death, inhibition of MAPK, PI3K/AKT, NF‐κB signaling pathways to induce apoptosis and inhibition of cell proliferation. Tomatine: activates caspase apoptosis signaling pathway, regulates PI3K/AKT pathway, regulates NF‐κB pathway, regulates PERK/eIF2α pathway; α‐Solanine inhibits tumor cell invasion, α‐Tomatine blocks cell cycle progression inhibition, regulates NF‐κB signaling pathway to induce apoptosis. However, most of the above conclusions come from in vitro experimental results and lack validation from in vivo animal models as well as clinical data. Whether alkaloidal active ingredients can provide evidence for SNL as a natural anticancer drug requires further research.

## 4 SAPONINS

Saponins are the most important class of steroids in SNL, and the anticancer potential of saponins has been summarized in the past. Olusola Olalekan Elekofehinti et al. summarized the classification of saponins and the mechanism of anticancer action for the^120^; Vakili SA et al. analyzed the mechanism of anticancer effects of saponin constituents by stimulating p53 expression^121^; Mingtao Zhu et al. summarized the anticancer mechanisms of saponin‐like components isolated from Chinese herbs^122^; Paulina Koczurkiewicz et al. summarize the potential of triterpenoid saponins in cancer therapy^123^;Shuli Man et al. summarized the research on saponins as active ingredients against cancer.^124^ This part mainly complements the mechanism of action of the saponin‐like components with anticancer activity isolated from SNL (Table 6).

**TABLE 6:** Anti‐tumor mechanism of saponins.

Cancer	Compound	Cell/Animal	Mechanisms	Bibliography
Lung cancer	Formosanin C	NCI‐H460 cells	Cellular autophagy associated with activation of JNK activity and inhibition of the PI3K/AKT/mTOR pathway	^125^
	β‐sitosterol	A549 cells	Mitochondria‐mediated apoptosis	^126^
	Daucosterol	A549 cells	Binding of DS to TrxR interfered with cellular redox homeostasis, activated the caspase‐3 apoptosis signaling pathway, and up‐regulated the level of phosphorylation of the tumor suppressor protein p53	^127^
Liver cancer	Daucosterol	HepG2 cells, SMMC‐7721 cells	Daucosterol inhibits migration and invasion of HCC cells through the Wnt/β‐catenin signaling pathway	^128^
	β‐sitosterol	HepG2 cells, Huh7 cells	Activation of caspase‐3 and − 9 activity, induction of apoptosis	^129^
	uttroside B	HepG2 cells	Regulation of MAPK and mTOR signaling pathways, activation of caspase pathway to induce apoptosis	^130^
Kidney cancer	Degalactotigonin	786‐O cells, A498 cells	Phosphorylation‐activated LATS1/2 induces YAP retention in the cytoplasm of RCC cells and blocks the interaction between YAP and TEAD1 to inhibit YAP mRNA expression and reduce YAP target gene expression	^132^
Pancreatic cancer	Degalactotigonin	PANC1 cells	reduced the level of EGF‐induced EGFR phosphorylation and inhibited phosphorylation of the downstream signaling molecules AKT (Ser 473) and ERK (Thr 202/Tyr 204).	^134^
	β‐sitosterol	MIA‐PaCa‐2 cells, BXPC‐3 cells	Downregulation of key molecules in the EMT pathway and inhibition of the AKT/GSK‐3 signaling pathway	^135^
Prostate cancer	Daucosterol	PC3 cells, LNCap cells	Activation of the jnk signaling pathway induces cellular autophagy	^136^
Osteosarcoma	Degalactotigonin	U2OS cells, HOS cells, MG‐63 cells	Promotion of GSK3β phosphorylation level inhibits GSK3β activity and hinders Gli1 expression	^137^

### 4.1 Lung cancer

Formosanin C is a biologically active steroidal saponin analog isolated from SNL. Treatment of human large‐cell lung cancer cells (NCI‐H460) with different concentrations of Formosanin C for 24 h up‐regulated the autophagy marker LC3‐II and key protein Beclin‐1 in a dose‐dependent manner. Formosanin C up‐regulated p‐JNK while down‐regulating p‐PI3K, p‐AKT, and p‐mTOR. Meanwhile, the co‐treatment with autophagy inhibitor chloroquine (CQ) induced JNK, PI3K, AKT, and mTOR phosphorylation to normal, suggesting that inhibiting NCI‐H460 cell proliferation by formosanin C through autophagy activation may be related to JNK activity activation and PI3K/AKT/mTOR pathway inhibition.^125^

Tamilselvam et al.^126^ found that β‐sitosterol treatment of A549 cells for 72 h significantly inhibited cell growth with an IC50 value of 24.7 μM and had no effect on normal human lung cells. This may be because β‐sitosterol interacts with Cys32 and Leu409 residues on Thioredoxin (Trx1) and Thioredoxin reductase (TrxR1) through hydrogen bonding, respectively. This interaction affects cellular redox homeostasis, promotes ROS accumulation, induces cellular DNA damage, and up‐regulates p53/p21 protein, triggering and activating cellular DNA damage. The p53/p21 protein up‐regulation activated and triggered mitochondria‐mediated apoptosis in A549 and NCI‐H460 cells.

Non‐small cell lung cancer is a common type of lung cancer, and daucosterol (DS) is a steroidal saponin compound widely found in the plant Lobelia. DS exhibited growth inhibitory effects on A549 cells with an IC50 value of 20.9 μM. The possible mechanism of action is that DS interacted with TrxR, disrupting cellular redox homeostasis. This disruption triggered oxidative stress‐mediated apoptosis, including the caspase‐apoptosis signaling pathway activation and up‐regulating p53 phosphorylation.^127^

### 4.2 Liver cancer

DS treatment of HepG2 and SMMC‐7721 cells down‐regulated β‐catenin and p‐β‐catenin. Co‐treatment of DS with SB‐216763, a Wnt signaling pathway inhibitor, attenuated the effects of DS on hepatocellular carcinoma cells. These results suggest DS may inhibit HCC cell migration and invasion through the Wnt/β‐catenin signaling pathway.^128^

Tuong et al. found that β‐sitosterol dose‐dependently inhibited HepG2 and Huh7 cell proliferation, with IC50 values of 0.017 and 0.021 μM, respectively, which may be related to activating caspase‐3/9 and inducing apoptosis by β‐sitosterol.^129^

NATH et al. found that uttroside B exhibited significant cytotoxicity against HepG2 cells, with an IC50 value of 500 μM, significantly reducing the tumor volume in xenograft tumor model mice. In vitro experiments revealed that treatment with uttroside B (500 μM) down‐regulated JNK, p38, and p42/44 (ERK1/2) phosphorylation and inhibited Phorbol 12‐myristate 13‐acetate (PMA)‐induced phosphorylation. Moreover, mTOR phosphorylation was reduced in a time‐dependent manner in HepG2 cells. These suggest that uttroside B may exert anti‐tumor effects by modulating the MAPK and mTOR signaling pathways, besides a dose‐dependent up‐regulating cleaved‐caspase 7/8/9.^130^

### 4.3 Kidney cancer

Degalactotigonin (DGT) is an SNL‐isolated steroidal glycoside with significant cytotoxicity.^131^ WANG et al. found that DGT inhibited the proliferation of renal cell carcinoma cell lines (786‐O and A498 cells) with IC50 values of 7.52–10.21 μM. The anti‐tumor mechanism of DGT is related to the key effector protein, yes‐associated protein (YAP), in the Hippo pathway. DGT induces YAP retention in the renal cell carcinoma cell cytoplasm by activating LATS1/2 protein phosphorylation and blocks YAP‐TEAD1 interaction to down‐regulate YAP and its target genes (associated with the Hippo pathway). The expression of YAP and its target genes AMOTL1, AMOTL2, AXL, CTGF, and CYR61 was reduced in DGT‐treated 786‐O and A498 cells compared to untreated control cells, therefore inhibiting cancer cell proliferation and inducing apoptosis.^132^

### 4.4 Pancreatic cancer

Epidermal Growth Factor Receptor (EGFR) and its family members have a regulatory role in tumor proliferation.^133^ Treatment of PANC‐1 cells with different concentrations (0, 1, 3, 10, and 30 μM) of DGT significantly reduced EGF‐induced phosphorylation levels of EGFR and inhibited in a concentration‐dependent manner the downstream signaling molecules AKT (Ser 473) and ERK (Thr 202/Tyr 204) phosphorylation. Additionally, 0.3 μM of DGT significantly induced cell cycle arrest in the G0/1 phase, inhibiting the growth effect on the proportion of S‐phase cells after EGF treatment. This suggests that DGT induces apoptosis and cell cycle arrest in pancreatic cancer cells by inhibiting the EGFR signaling pathway.^134^

Treatment of human pancreatic cancer cells (MIA‐PaCa‐2) and human in situ pancreatic adenocarcinoma cells (BXPC‐3 cells) with different β‐sitosterol concentrations (0, 125, 250, and 500 μM) inhibited cancer cell migration and invasion by regulating EMT pathway key molecules (dose‐dependently down‐regulating Snail and vimentin proteins and up‐regulating E‐cadherin protein), thereby exerting anti‐pancreatic cancer activity by inactivating the AKT/GSK‐3 signaling pathway and regulating apoptosis.^135^

### 4.5 Prostate cancer

Gao et al.^136^ found that DS treatment of human prostate cancer cells (PC3 and LNCap cells) for 48 h increased c‐Jun N‐terminal kinase (JNK) phosphorylation and induced cellular autophagy. Meanwhile, the autophagy inhibitor 3‐MA and the JNK‐specific inhibitor SP600125 could inhibit DS‐induced cellular autophagy, suggesting that DS partly inhibits prostate cancer growth by activating the JNK signaling pathway and inducing autophagy.

### 4.6 Osteosarcoma

Zhao et al.^137^ found that DGT inhibited the proliferation of human osteosarcoma cell lines (U2OS, HOS, and MG‐63 cells) in vitro, with IC50 values of 12.91–31.46 μM. DGT treatment down‐regulated GLI Family Zinc Finger 1 (Gli1) in a dose‐dependent manner while up‐regulating GSK3β phosphorylation to inhibit GSK3β activity, thus inhibiting cell viability and invasion. Cell viability and invasion were partially restored when transfected into GSK3β knockdown ZOS cells using the overexpression of the Gli1 plasmid. This suggests that DGT may inhibit osteosarcoma cell activity by suppressing GSK3β/Gli1 activation.

In summary, among several major saponin active ingredients of SNL, Formosanin C inhibited the PI3K/AKT/mTOR pathway to induce cellular autophagy; β‐sitosterol induced mitochondria‐mediated apoptosis, activated the caspase pathway to activate apoptosis, and regulated the AKT/GSK‐3 signaling pathway to inhibit cell migration. Binding of Daucosterol to TrxR interferes with cellular redox homeostasis to activate the caspase‐3 apoptosis signaling pathway, and inhibits migration and invasion of HCC cells through the Wnt/β‐linker protein signaling pathway. Activation of the jnk signaling pathway induces cellular autophagy; uttroside B activation of caspase pathway to induce apoptosis. Degalactotigonin inhibits the EGFR signaling pathway to induce apoptosis and cell cycle arrest, and the Hedgehog/Gli1 Pathway to inhibit tumor cell migration and invasion. In order to be able to make a case for SNL as a natural anticancer drug, the long‐term effects, bioavailability and anticancer activity of these components in different tumor cells need to be investigated, and also supplemented with in vivo animal models to confirm their efficacy and safety.

## 5 POLYSACCHARIDES

Polysaccharide components have been less studied due to reasons such as difficulty in extraction, complexity of composition, and most of the active components have not been identified, etc. This part of the study focuses on the anti‐tumor mechanism of action of polysaccharides isolated from Lobelia (Table 7).

**TABLE 7:** Anti‐tumor mechanism of polysaccharides.

Cancer	Compound	Cell/Animal	Anticancer effect	Mechanism	Bibliography
Lung cancer	snlp‐1	Female C57 BL/6 mice, RAW264.7 mouse macrophages and Lewis lung cancer (LLC) cell line		Up‐regulation of mRNA and protein expression of TLR4, MyD88, TRAF6, p‐NF‐κB, p‐c‐Jun induces macrophages to release NO, TNF‐α and IL‐6	^138^
Breast cancer	SN‐ppF3	Female BALB/c mice Mouse breast cancer cell line 4 T1	Improvement of the immune response	Increased levels of cytokines (TNF‐α, IFN‐α, and IL‐4) and enhanced K‐cell, CD8+ T‐cell, and macrophage activity	^140^
Cervical cancer	snlp	Female Kunming mice U14	Induction of apoptosis	Downregulated Bcl‐2 and mutant p53 gene expression and upregulated Bax	^141^
	snlp1a	Female Kunming mice U14	Improvement of the immune response	Restoration of normal Bcl‐2/Bax ratio and increased activity of CD4+/CD8+ T‐lymphocyte subsets	^29^

### 5.1 Lung cancer

Pu et al.^138^ isolated a homogeneous polysaccharide from SNL (SNLP‐1) and analyzed its effects on macrophages and Lewis lung carcinoma (LLC) tumor model mice. The results showed that SNLP‐1 significantly up‐regulated mRNAs and proteins of key nodes in the TLR4 signaling pathway, including TLR4, MyD88, TRAF6, p‐NF‐κB, p‐c‐Jun, and induced NO, TNF‐α, and IL‐6 release from macrophages. In vivo results showed that after 20 days of gavage administration of SNLP‐1 (4 mg/kg/day), macrophages in the LLC tumor model mice significantly down‐regulated NO, TNF‐α, and IL‐6, reducing tumor volume and weight. Additionally, SNLP‐1 significantly up‐regulated cytokine levels, including IL‐2, IFN‐γ, and TNF. Altogether, SNLP‐1 can enhance immune activity and play an anti‐tumor role by regulating the TLR4‐mediated MyD88‐dependent signaling pathway.

### 5.2 Breast cancer

Breast cancer is the most common female cancer in clinical practice,^139^ and SN‐ppF3, a polysaccharide component extracted from SNL, inhibits mouse breast cancer. The in vivo experiments showed that oral administration of 250 and 500 mg/kg of SN‐ppF3 for 10 days inhibited tumor growth in 54% and 65% of mice, respectively. The analysis of cytokine levels showed that TNF‐α was elevated by ~14% compared with the control group and up‐regulated other cytokines, IFN‐γ (~22%) and IL‐4 (~12%). Immunofluorescence assay showed that the fluorescence intensity of natural killer cells, CD8^+^ T cells, and macrophages was significantly enhanced after SN‐ppF3 treatment, suggesting that SN‐ppF3 exerts its anti‐tumor effects by enhancing the immune response.^140^

### 5.3 Cervical cancer

Cervical cancer is a highly prevalent and lethal disease in women worldwide, with ~600,000 cases and 300,000 deaths worldwide in 2020.^1^ SNLP induces apoptosis in cervical cancer lines (U14 cells). In vivo studies showed that 180 and 360 mg/kg SNLP administered by gavage for 12 consecutive days significantly reduced tumor weight and volume in xenograft tumor‐bearing mice. Moreover, SNLP significantly down‐regulated Bcl‐2 and mutant p53 proteins while up‐regulating Bax, suggesting that SNLP exerts anti‐tumor effects by inducing apoptosis through apoptotic signaling regulation.^141^

Li et al.^29^ isolated three homogeneous polysaccharides, SNLP‐1a/b/c, from SNL, all of which reduced tumor weights in a mouse cervical cancer model. The inhibitory effect on tumor growth after treatment with SNLP‐1a was the most significant. Immunohistochemical staining of thymus tissue sections showed that SNLP‐1a could restore their normal Bcl‐2/Bax ratio and reduce the damage caused by tumor invasion. Additionally, the ratio of CD4^+^/8^+^ T‐lymphocyte subpopulation was increased from 0.77 to 1.97 and 2.62, respectively. SNLP‐1a exerts anti‐tumor activity by inhibiting tumor‐induced apoptosis of thymus lymphocytes in vivo and enhancing the immune response.

In summary, among the several polysaccharide components isolated from SNL, SNLP‐1 promotes immune activity through the activation of MyD88‐dependent signaling pathway by TLR4, and the rest of the polysaccharide components also exert anti‐tumor activity mainly through the pathways of enhancing immunity and inducing apoptosis, etc. Due to factors such as the instability and difficulty of isolation of the polysaccharide components, which have hindered the study of this kind of components, more research is needed to confirm the anti‐tumor activity of the polysaccharide components in SNL.

## 6 POLYPHENOLS

Several studies have been conducted to show the inhibitory effects of phenolic acid components contained in SNL, including GA, ferulic acid, caffeic acid, and quercetin, on breast, lung, liver, and prostate cancers. Gupta Ashutosh et al.^142^ and Marjorie Reyes‐Farias et al.^143^ described the anti‐tumor mechanism of ferulic acid and quercetin, respectively. Ashrafizadeh Milad et al.^144^ and Alam Manzar et al.^145^ summarized the mechanism of action of GA and caffeic acid in cancer therapy, respectively. Jiang et al.^146^ outlined the potential role of GA as an anti‐tumor drug. Kuar et al.^147^ analyzed the characteristics and anti‐tumor effects of ferulic acid as a multi‐target therapeutic agent. Shukla et al.^148^ described the novel delivery system of ferulic acid for its anti‐tumor effects. Bastidas et al.^149^ summarized the important studies of caffeic acid and its derivatives in cancer therapy. This section will add to the recent studies on polyphenolic components extracted from SNL (Table 8).

**TABLE 8:** Anti‐tumor mechanism of polyphenols.

Cancer	Compound	Cell/Animal	Mechanism	Bibliography
Lung cancer	Gallic acid	A549 cells	Inhibition of PI3K/Akt pathway	^150^
	Quercetin	A549 cells	Phosphorylation of EGFR and Akt and expression of EGFR	^151^
	Quercetin	A549 cells	Novel Drug Delivery Formulations Enhance Drug Targeting	^152^
	Gallic acid	A549 cells/PC9 cells	Inhibition of the Hippo‐YAP signaling pathway enhances IH‐induced caspase‐3‐mediated apoptosis	^153^
Liver cancer	SNPE	HepG2 cells	Regulation of the CDC25 family and CDK1 activity induced cell cycle arrest	^154^
Lymphoma	Aqueous extract of SN leaves	AU565 cells	Low dose induced autophagy, high dose induced autophagy and apoptosis	^31^
	Quercetin	MCF‐7 cells, MDA‐231 cells	Induction of TFEB nuclear translocation, activation of lysosomal activity, promotion of ferritin degradation and induction of cellular iron death	^155^
	Gallic acid	MDA‐MB‐231 cells	Increases cell membrane permeability and cytochrome c levels, activates caspase‐8 and caspase‐9 activity	^156^
Gastric cancer	Gallic acid	GPL model cells established by GES‐1 cells	Inhibition of the Wnt/β‐catenin signaling pathway and the EMT pathway	^157^
Colorectal cancer	Quercetin	LI32 cells, HCT 116 cells, COLO 320 cells, COLO 205 cells	Regulation of the expression of anti‐aging genes SIRT‐6 and Klotho	^158^
		HCT‐116 cells	Inhibition of Resistin‐induced TLR4‐mediated up‐regulation of NLRP3 expression with increased ERK phosphorylation levels enhances sensitivity to 5‐Fluorouracil	^159^
		RKO cells, SW480 cells, and HCT116 cells	Regulation of the JNK signaling pathway	^160^
		HT‐29 cells	Novel Drug Delivery Formulations Enhance Drug Targeting	^161^
Prostate cancer	Quercetin	CWR22Rv1 cells, VCaP cells	Inhibition of ARv7 expression and down‐regulation of ARv7‐mediated circNHS/miR‐512‐5p/XRCC5 signaling	^162^
Squamous cell carcinoma of the tongue	Quercetin	The human tongue SCC‐derived cell line SAS (JCRB0260)	Promotes JNK activation and regulates ERK1/2 and GSK3‐α/β‐mediated mitochondria‐dependent apoptotic signaling pathways	^163^

### 6.1 Lung cancer

Ko et al.^150^ found that GA inhibited cell colonization and tumor spheroid formation in A549 cells. Additionally, tumor volume and weight decreased significantly after 4 weeks of intraperitoneal administration to xenograft model mice. The anti‐tumor mechanism of GA involved inhibiting the PI3K/Akt pathway by down‐regulating PI3K protein while inducing Akt phosphorylation at Ser473 and Thr308. Ganthala et al.^151^ developed novel TPGS‐MA‐CH solid lipid nanoparticles that enhanced drug targeting and bioavailability to tumor tissues by significantly down‐regulating EGFR and Akt phosphorylation and EGFR expression in A549 cells. Moreover, Mahdi et al.^152^ developed a novel carboxymethyl cellulose‐based hydrogel with Core‐Shell Fe_3_O_4_@SiO_2_ nanoparticles that improved the cytotoxicity and antiproliferative effect of quercetin on A549 cells. Wang et al.^153^ found that GA, combined with Icotinib hydrochloride (IH), enhanced A549 and PC9 cell sensitivity to IH, inducing apoptosis. Additionally, co‐treatment of IH and GA significantly increased the YAP phosphorylation in A549/PC9 cells and caspase‐3 mRNA expressions while downregulating YAP. This suggests that GA and IH combination could enhance IH‐induced apoptosis by inhibiting the Hippo‐YAP signaling pathway to enhance IH‐induced caspase‐3‐mediated apoptosis.

### 6.2 Liver cancer

SNPE treatment activated cleaved‐caspase‐3/8/9, significantly downregulating apoptosis‐related proteins Bcl‐2 and Bid. This indicates that SNPE induces apoptosis in the HepG2 cells through caspase activation and Bcl‐2 family regulation. Furthermore, the increased SNPE levels increased the blocked HepG2 cells in the G2/M phase in a dose‐dependent manner, significantly reduced the protein levels of the CDC25 family (including CDC25A/B/C, which is important in cell cycle regulation), and significantly decreased the formation of cell division cycle 2‐like 6 (CDK1) and Cyclin B complexes with the SNPE amount is significantly reduced. This suggests that SNPE may induce cell cycle arrest in HepG2 cells by regulating the activity of the CDC25 family and CDK1.^154^

### 6.3 Breast cancer

The aqueous extract of SN leaves has significant cytotoxic effects on human breast cancer cells (AU565). After treatment with different doses (0.028 and 0.113 μg/mL) of aqueous extract of SN leaves, the expression of autophagy marker LC‐3 protein was increased by the low‐dose treatment, reaching the peak value at 6–12 h. Meanwhile, after 48 h of the high‐dose treatment, LC‐3 protein and apoptosis marker c‐PARP were up‐regulated. This indicates that SN leaf aqueous extract exerts anti‐tumor effects by mediating different mechanisms in a dose‐dependent manner.^31^

An et al.^155^ found that quercetin induced transcription factor EB (TFEB) nuclear translocation, activated lysosomal activity, promoted ferritin degradation, and increased intracellular ferric ions, inducing iron death in breast cancer cells. Safa et al.^156^ found that GA had significant cytotoxicity against MDA‐MB‐231 breast cancer cells, with an IC50 of 0.2578 μM. GA increased cell membrane permeability, cytochrome c levels, and caspase‐8/9 activities while significantly decreasing mitochondrial membrane permeability and inducing apoptosis. This suggests that GA may be a potential therapeutic agent for triple‐negative breast cancer.

### 6.4 Stomach cancer

Liao et al.^157^ induced GES‐1 cells with N‐methyl‐N′‐nitro‐N‐nitrosoguanidine (MNNG) to establish gastric precancerous lesions (GPL) model cells, revealing that GA down‐regulated EMT pathway‐related proteins, N‐cadherin and vimentin while up‐regulating E‐cadherin. Additionally, GA down‐regulated the key proteins of the Wnt/β‐catenin signaling pathway, Wnt 10B and β‐catenin, and its downstream protein Cyclin D1. Meanwhile, the Wnt activator, Wnt agonist 1, could reverse this regulatory effect of GA on GPL model cells. This suggests that GA attenuates GPL by inhibiting the Wnt/β‐catenin and EMT signaling pathways.

### 6.5 Colorectal cancer

In their study, Bhatiya et al^158^ observed that Quercetin effectively suppressed the proliferation of colon cancer cells by modulating the expression of the anti‐aging genes SIRT‐6 and Klotho. Additionally, Lee et al^159^ demonstrated that Quercetin inhibited the Resistin‐induced TLR4‐mediated NLRP3 expression and ERK phosphorylation levels in HCT‐116 cells, thereby enhancing the susceptibility of human colon cancer cells to 5‐Fluorouracil. Quercetin was observed to exhibit a substantial inhibition of cell invasion, while also activating the JNK signaling pathway. Additionally, the antiproliferative impact on colonic cancer cells induced by SP600125, a JNK pathway inhibitor, was effectively reversed by quercetin. These findings strongly imply that quercetin exerts its inhibitory effects on the migration and invasion of colon cancer cells through the modulation of the JNK signaling pathway.^160^ Liu et al^161^ developed a conatumumab decorated, reactive oxygen species sensitive irinotecan prodrug and quercetin co‐loaded nanostructured lipid carriers, which significantly inhibited the growth of HT‐29 cells and enhanced drug targeting without systemic without systematicity.

### 6.6 Prostate cancer

The study revealed that radiotherapy had the effect of increasing the expression of ARv7 in CWR22Rv1 and VCaP cells. This increase in ARv7 expression was found to potentially decrease the sensitivity of these cells to radiation by upregulating the expression of circRNA NHS. It was further observed that circRNA NHS binds to miR‐512‐5p, leading to an increase in the protein expression of XRCC5. Additionally, the administration of Quercetin was found to significantly reduce the expression of ARv7 at both the mRNA and protein levels in CWR22Rv1 and VCaP cells. Furthermore, Quercetin was observed to down‐regulate the ARv7‐mediated circNHS/miR‐512‐5p/XRCC5 signaling pathway, thereby enhancing the radiosensitivity of prostate cancer. This finding was reported in a study by.^162^

### 6.7 Squamous cell carcinoma of the tongue

Huang et al^163^ discovered that quercetin facilitated JNK activation in order to regulate the ERK1/2 and GSK3‐α/β‐mediated mitochondria‐dependent apoptotic signaling pathway, thereby inducing apoptosis in squamous cell carcinoma of the tongue. This conclusion was primarily derived from the observation that quercetin notably elevated the protein expression levels of p‐ERK, p‐JNK1/2, and p‐GSK3‐α/β in SAS cells and exerted its apoptotic effects by modulating the expression of proteins associated with the mitochondrial apoptotic signaling pathway.

In summary, Quercetin can regulate circNHS/miR‐512‐5p/XRCC5 signaling pathway, mitochondria‐dependent apoptosis signaling pathway, JNK signaling pathway, Regulation of the expression of anti‐aging genes SIRT‐6 and Klotho, induction of cellular iron death, induction of autophagy and apoptosis, and enhancement of drug sensitivity and targeting; gallic acid modulates the PI3K/Akt pathway, the Hippo‐YAP signaling pathway, activates the caspase pathway, and inhibits the Wnt/β‐linker protein signaling pathway and the EMT pathway. In order to better establish SNL as a safe and effective natural anticancer drug, more mechanistic studies, in vivo animal model experiments and human clinical studies are needed.

## 7 SUMMARY AND DISCUSSION

This paper summarizes the results of recent studies on the chemical composition of SNL and describes the anti‐tumor mechanism of its active components. A variety of active components such as sterols and saponins, alkaloids, polysaccharides, and phenolic acids extracted from SNL possess anti‐tumor activity. These active components exert their anti‐tumor activities by regulating various signaling pathways to induce apoptosis, cell autophagy, iron death of tumor cells, inhibition of tumor cell invasion and migration, and other events. Most of the active ingredients induced apoptosis mainly by regulating the caspase pathway and mitochondrial apoptosis pathway, and some of the active ingredients could affect the cellular redox homeostasis to activate the caspase‐3 apoptosis signaling pathway. In addition, by enhancing the ubiquitination degradation of SLC1A1 or disrupting cellular redox homeostasis, they can activate iron death in tumor cells. The active ingredients can regulate the Wnt/β‐cyclin signaling pathway and EMT pathway to inhibit the migration or invasion of tumor cells. Some active ingredients inhibit tumor cell growth by enhancing immune response, improving drug sensitivity and targeting. In addition, some active ingredients inhibit tumor cell growth by regulating certain lncRNA or miRNA‐related pathways involved.

Current research on SNL has focused on cellular and animal models, and the actual effects on the human body have not yet been fully demonstrated and need to be further investigated in conjunction with the available clinical evidence. In addition, most studies have investigated the anti‐tumor effects of individual components, and the synergistic effects of different components on anti‐tumor need to be investigated. The active components of SNL can improve the efficacy of clinical chemotherapeutic drugs and weaken the drug resistance of cancer cells. Therefore, the establishment of a new delivery vehicle to improve drug bio‐availability and drug targeting to tumor tissues is an important direction for the development of potential anti‐tumor drugs. In addition, SNL contains potentially toxic compounds, and better quality control standards need to be established to ensure the safety of SNL.

**FIGURE 5:**
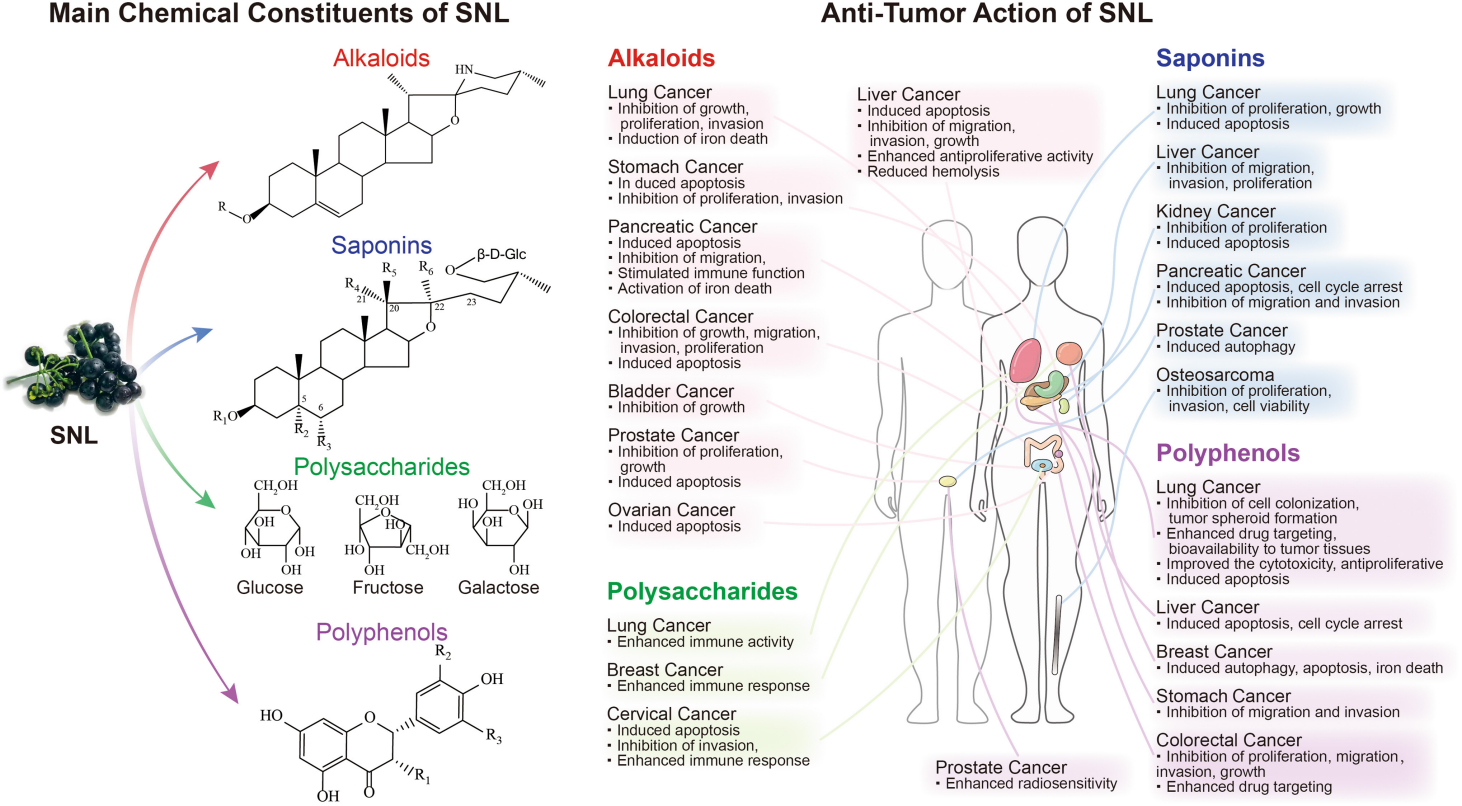
Main chemical constituents of SNL.

In summary, SNL, as a widely distributed natural drug, has potential therapeutic effects on a wide range of tumors, and the active components of SNL inhibit tumor development through a multi‐pathway and multi‐target mechanism of action. This paper describes the active components and mechanism of action of SNL, revealing the great potential and broad research prospects of SNL as a potential anti‐tumor drug (Figure 5).

## AUTHOR CONTRIBUTIONS

**Zhen‐duo Zhao:** Writing – original draft (lead). **Cheng Hu:** Writing – original draft (supporting). **Ling Li:** Conceptualization (equal); writing – review and editing (supporting). **Jia‐qi Zhang:** Conceptualization (equal); writing – review and editing (lead). **Li‐chao Zhang:** Conceptualization (lead); writing – review and editing (equal).

## FUNDING INFORMATION

This study was supported by grants from the National Natural Science Foundation of China (Nos. 81973551 and 82204686), the Science and Technology Commission of Shanghai Municipality (No.21ZR1460400), Future Plan for Traditional Chinese Medicine Inheritance and Development of Shanghai Municipal Hospital of Traditional Chinese Medicine (WLJH2021ZY‐ZYY007; WL‐HBBD‐2021001K), and the Health Commission of Shanghai Municipality (ZY(2021‐2023)‐0203‐04).

## CONFLICT OF INTEREST STATEMENT

The authors have no conflict of interest.

## Data Availability

Data sharing not applicable—no new data generated.
